# Neurorehabilitation Through Synergistic Man-Machine Interfaces Promoting Dormant Neuroplasticity in Spinal Cord Injury: Protocol for a Nonrandomized Controlled Trial

**DOI:** 10.2196/41152

**Published:** 2022-09-13

**Authors:** Alkinoos Athanasiou, Konstantinos Mitsopoulos, Apostolos Praftsiotis, Alexander Astaras, Panagiotis Antoniou, Niki Pandria, Vasileia Petronikolou, Konstantinos Kasimis, George Lyssas, Nikos Terzopoulos, Vasilki Fiska, Panagiotis Kartsidis, Theodoros Savvidis, Athanasios Arvanitidis, Konstantinos Chasapis, Alexandros Moraitopoulos, Kostas Nizamis, Anestis Kalfas, Paris Iakovidis, Thomas Apostolou, Ioannis Magras, Panagiotis Bamidis

**Affiliations:** 1 Medical Physics and Digital Innovation Lab School of Medicine, Faculty of Health Sciences Aristotle University of Thessaloniki Thessaloniki Greece; 2 Computer Science Department Division of Science and Technology American College of Thessaloniki Thessaloniki Greece; 3 Department of Physiotherapy International Hellenic University Thessaloniki Greece; 4 Department of Design, Production and Management University of Twente Enschede Netherlands; 5 Laboratory of Fluid Mechanics and Turbo-machinery Department of Mechanical Engineering Aristotle University of Thessaloniki Thessaloniki Greece; 6 Second Department of Neurosurgery Ippokrateio General Hospital Aristotle University of Thessaloniki Thessaloniki Greece

**Keywords:** body-machine interface, brain-computer interface, neural rehabilitation, serious games, spinal cord injury, wearable robotics

## Abstract

**Background:**

Spinal cord injury (SCI) constitutes a major sociomedical problem, impacting approximately 0.32-0.64 million people each year worldwide; particularly, it impacts young individuals, causing long-term, often irreversible disability. While effective rehabilitation of patients with SCI remains a significant challenge, novel neural engineering technologies have emerged to target and promote dormant neuroplasticity in the central nervous system.

**Objective:**

This study aims to develop, pilot test, and optimize a platform based on multiple immersive man-machine interfaces offering rich feedback, including (1) visual motor imagery training under high-density electroencephalographic recording, (2) mountable robotic arms controlled with a wireless brain-computer interface (BCI), (3) a body-machine interface (BMI) consisting of wearable robotics jacket and gloves in combination with a serious game (SG) application, and (4) an augmented reality module. The platform will be used to validate a self-paced neurorehabilitation intervention and to study cortical activity in chronic complete and incomplete SCI at the cervical spine.

**Methods:**

A 3-phase pilot study (clinical trial) was designed to evaluate the NeuroSuitUp platform, including patients with chronic cervical SCI with complete and incomplete injury aged over 14 years and age-/sex-matched healthy participants. Outcome measures include BCI control and performance in the BMI-SG module, as well as improvement of functional independence, while also monitoring neuropsychological parameters such as kinesthetic imagery, motivation, self-esteem, depression and anxiety, mental effort, discomfort, and perception of robotics. Participant enrollment into the main clinical trial is estimated to begin in January 2023 and end by December 2023.

**Results:**

A preliminary analysis of collected data during pilot testing of BMI-SG by healthy participants showed that the platform was easy to use, caused no discomfort, and the robotics were perceived positively by the participants. Analysis of results from the main clinical trial will begin as recruitment progresses and findings from the complete analysis of results are expected in early 2024.

**Conclusions:**

Chronic SCI is characterized by irreversible disability impacting functional independence. NeuroSuitUp could provide a valuable complementary platform for training in immersive rehabilitation methods to promote dormant neural plasticity.

**Trial Registration:**

ClinicalTrials.gov NCT05465486; https://clinicaltrials.gov/ct2/show/NCT05465486

**International Registered Report Identifier (IRRID):**

PRR1-10.2196/41152

## Introduction

Spinal cord injury (SCI) constitutes a major sociomedical problem, impacting approximately 0.32-0.64 million people each year worldwide; particularly, it impacts young individuals, causing long-term, often irreversible disability [1]. The sensorimotor networks of patients with SCI and healthy individuals share similar connectivity patterns, but new functional interactions have been identified as unique to the sensorimotor networks of patients with SCI [2-4] and can be attributed to both adaptive and maladaptive organization effects after the injury. The importance of such phenomena both as possible prognostic factors and as contributors to patient rehabilitation remains unspecified as of yet. The exact underlying neurophysiological process and the extent to which this is modulated by higher-order interactions are also not fully understood [5]. By contrast, researchers have recently demonstrated for the first time partial neurological recovery 5-10 years after complete SCI through groundbreaking neurorehabilitation protocols [6]. The investigators used rich visual and tactile feedback, virtual reality environments, a brain-computer interface (BCI)–controlled exoskeleton, and robotic actuators, as well as documented plasticity effects at the cortical level to support their theory [6-8]. The authors found that patients experienced integration of virtual legs into their body schema and proceeded to develop a multistage neurorehabilitation protocol for patients with chronic SCI. While the original aim was to explore a long-term body-machine interface (BMI)–based protocol for patients with SCI that helps them regain the ability to walk autonomously using brain-controlled exoskeletons, unexpectedly patients with chronic SCI experienced a significant clinical improvement in their ability to perceive somatic sensations and exert voluntary motor control in dermatomes located below the injury. An electroencephalography (EEG) analysis revealed clear signs of cortical functional plasticity, suggesting, for the first time, that such long-term multimodal training may induce sensorimotor plasticity capable of triggering partial neurological recovery [9,10]. The importance of proper control scheme design in targeting neural plasticity during neural rehabilitation has also been demonstrated by invasive interfaces, such as spinal cord stimulation below the injury level, where novel findings suggest that reproducing natural central nervous system (CNS) input and output patterns below the injury level can lead to long-term functional improvement, even without stimulation, for select patients with chronic SCI [11].

Residual communication between brain and spinal cord plays an important role in possible neurorehabilitation, as even in complete injuries one-fourth of nerve fibers crossing the injury level are functionally intact. As such, retraining CNS circuits and promoting plasticity to restore body functions have been recognized among key principles of spinal cord repair by the US National Institute of Neurological Disorders and Stroke (US NIH/NINDS). Nonetheless, existing literature does not yet portray with precision the pathophysiological process and effect of SCI on CNS and the sensorimotor networks [2,12-14]. Studies needed to address this issue should consider identifying specific questions to be answered through further investigation: (1) how and why reorganization of CNS networks are established, (2) how this reorganization evolves in time with respect to the severity and chronicity of the injury, (3) when can it be considered an adaptive or maladaptive evolution, and (4) how can it be promoted or prevented, respectively. The insight gained is expected to hold clinical relevance in preventing maladaptive plasticity after SCI through individualized neurorehabilitation, as well as in the design of assistive technologies for patients with SCI. While new assistive technologies, including robotics and spinal cord stimulation, have emerged as potential methods to replace lost mobility [15], it has been demonstrated that SCI impacts the whole CNS, inducing adaptive and maladaptive neuroplasticity. Multiple training modalities, immersive environments, and rich and versatile feedback (visual and tactile) in neurorehabilitation have demonstrated the ability to promote neural plasticity and induce partial neurological recovery in paraplegia even after chronic complete SCI.

The main hypothesis for the NeuroSuitUp clinical trial suggests that patients with chronic upper SCI can benefit from training in multiple man-machine modalities and present neurological improvements due to synergistically induced neuroplasticity. As such, the objective of NeuroSuitUp is to develop, pilot test, and optimize a platform based on multiple immersive man-machine interfaces offering rich feedback that include (1) visual motor imagery (VMI) training under high-density EEG recording, (2) mountable robotic arms controlled with wireless BCI, (3) a BMI consisting of wearable robotics jacket and gloves in combination with a serious game (SG) application and rich audiovisual stimuli, and (4) an augmented reality (AR) module. The platform will be used to validate a self-paced neurorehabilitation intervention and to study cortical activity in chronic complete and incomplete SCIs at the cervical spine.

## Methods

The components of the NeuroSuitUp platform, the protocol used for patient recruitment, the experimental procedures for the pilot testing, and the questionnaires and outcomes used in the study are described hereby.

### Overview of the NeuroSuitUp platform

The main components of the NeuroSuitUp platform have been selected to assemble an immersive platform consisting of multiple man-machine interfaces that present synergies in their use and implementation. The components consist of the following: (1) VMI training under high-density EEG recording, (2) mountable robotic arms controlled with wireless BCI, (3) BMI consisting of wearable robotics jacket and gloves in combination with an SG application, and (4) an AR module.

### Visual Motor Imagery Under High-Density Electroencephalography

**Figure 1:**
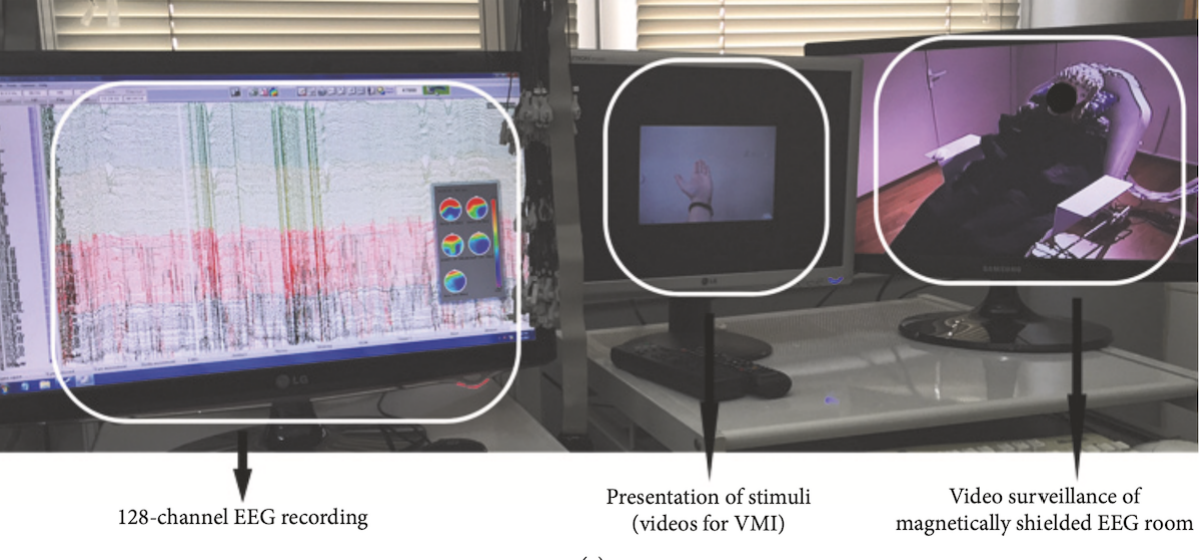
VMI under high-resolution EEG recording during presentation of multiple movements of the upper limb by patients with spinal cord injury in previous pilot experiment [3,16]. Figure from [16]. EEG: electroencephalography; VMI: visual motor imagery.

Participants during the 3 main sessions (initial, intermediate, final) will train their VMI by attempting multiple imaginary movements of the upper limbs (32 possible movements of the right and left upper extremities), and during walking imagery, while under high-density EEG recording (128 channels). The participants will watch age- and sex-matched upper limbs in a screen performing these motions while attempting to imagine that these are their own arms performing the movement. The torso and arms of the participants will be covered by a black sheet to facilitate registration of the presented extremities into their own body schema (Figure 1). This experimental setup has already been tested by the authors with patients with SCI to train motor-related imagery and pretrain mental strategies for kinesthetic BCI classification [3,4].

### Mountable Robotic Arms Controlled With a Wireless Brain-Computer Interface

**Figure 2:**
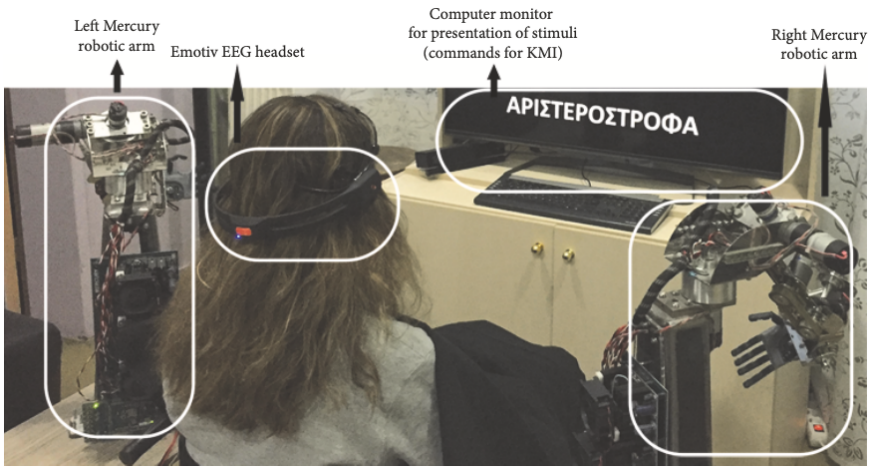
Pilot use of 8-degrees-of-freedom robotic arms by a 30-year-old patient with spinal cord injury with C6 tetraparesis, controlled by a commercial EEG-based brain-computer interface (Emotiv) through KMI [16,17]. Figure from [16]. EEG: electroencephalography; KMI: kinesthetic motor imagery.

Participants during the 3 main sessions (initial, intermediate, and final) will train in kinesthetic motor imagery (KMI) to control the anthropomorphic 8-degree-of-freedom robotic arms (Mercury 2.0) that have been developed by the Lab of Medical Physics & Digital Innovation. Control will be achieved by a commercial-level, wearable, wireless 14-channel EEG-based BCI (the Emotiv EPOC). This experimental setup has already been tested by the authors with patients with SCI [16,17], achieving high rates of BCI performance and high degree of user acceptance of the robotic system (Figure 2).

### A Body-Machine Interface Consisting of Wearable Robotics Jacket and Gloves in Combination With Serious Games Application

**Figure 3:**
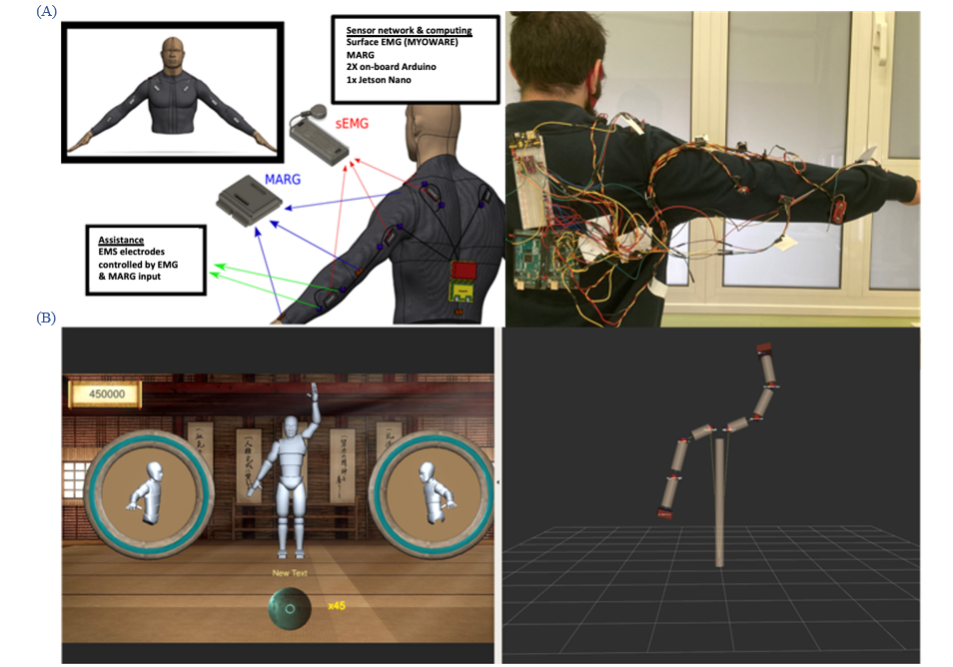
(A) Schematic of sensors layer and photo of the wearable prototype. (B) Unity game snapshot and corresponding pose in the robot operating system (ROS) rviz tool. Figure modified from [18]. EMG: electromyography; EMS: electrical muscle stimulation; sEMG: surface electromyography.

Our wearable device (wearable robotics jacket and gloves) was designed with the purpose of providing a real-time quantifiable evaluation of the wearers upper body kinematics and the accompanying biomechanical systems that enable them [15]. The device consists of 2 types of commercially available sensors, attached on the cloth of the wearable. Six 9-degrees-of-freedom magnetic acceleration rotation gravity sensors were placed on the links of 3D representation of the upper body kinematic chain. They are complemented by 14 surface electromyography (sEMG) sensors placed on the major muscle groups impacting the state of the joints in the aforementioned kinematic chain [18,19]. The information from the sensors is collected and transferred from an Arduino board to an Ubuntu-based computer, running a robot operating system environment. The data are then collected, with the information gathered by the inertial measurement units being used to calculate the angles of the joints where the connected links are attached. The calculated kinematic representation is then directed to a live visualization SG, created in Unity engine (Figure 3). Utilizing the live representation of the wearer’s kinematics, in combination with EMG activity from the muscles that directly impact the kinematic chain, the user can train on prescribed tasks in the SG, while receiving assistance in the form of surface electrical muscle stimulation (EMS) according to desirable difficulty level.

### Augmented Reality Module

This modality will use the Microsoft HoloLens 2.0 AR Headset and the Unity engine to project the NeuroSuitUp dojo-themed SG, as well as virtual robotic arms, and to provide the participants with an easy-to-use, readily accessible platform to train in task-specific movement of the arms (grasping, reaching) and different degrees of freedom while in an immersive AR platform [20]. Control will be provided by both EEG-BCI [21] and the BMI developed to train in an AR environment. The virtual avatar and arms will be fully anthropomorphic and motion will be fluid. The set up will also deploy an array of wearable sensors for physiological recording (heart rate, pressure, skin conductance) to assess affective parameters of neurorehabilitation.

### Ethics Approval

The study was approved by the Committee for Bioethics and Ethics of the School of Medicine, Aristotle University of Thessaloniki with the protocol number 188117/2022 in the Committee sitting 68/13-7-2022 following the application with the protocol number 165080/2022.

### Patient Population and Eligibility Criteria

Participants considered for recruitment are reviewed for the following eligibility criteria (inclusion and exclusion) by a member of the research team before enrollment.

#### Inclusion Criteria

The following criteria should be met by eligible participants before inclusion into the study:

- Participants (patients with SCI or healthy individuals) should be at least 14 years of age.
- Participants of the SCI group (complete or incomplete) should have a clinical diagnosis of SCI evaluated by the ASIA (American Spinal Injury Association) Impairment Scale (AIS).
- Participants of the SCI groups (complete or incomplete) should have sufficient documentation of the injury in neurological examination and a magnetic resonance imaging (MRI) scan of the injury level, as well as optional additional computed tomography or x-rays.
- Participants should be willing to follow the study protocol and procedures.
- Parents or legal guardian should voluntarily provide written consent for their child’s participation in the study, in case participants are under 18 years of age.

#### Exclusion Criteria

The following criteria will result in the exclusion of participants from the study:

- Any other neurological condition that has a possibility to significantly impact the neurological status of the participants or the ability to control a BCI or the neurophysiological recordings:
  traumatic brain injury
  CNS tumors
  multiple sclerosis
  amyotrophic lateral sclerosis
  Parkinson disease
  refractory epilepsy
- Participation during the last 3 months in another interventional study, the effects of which could impact this study’s observations.
- Other grave medical condition that could impact the participation or the safety of the participants:
  cardiac deficiency
  pulmonary deficiency
  hearing and visual impairments that can impact the participant’s understanding of the intervention and performance
  illegal drug use
  chronic alcoholism
- Parents who are not willing to provide written consent for their child’s participation in the study.

A “Participant Information and Consent Form” is used to comprehensively inform all participants regarding the procedures of the study, the purpose of the study, and potential risks and benefits. The approval of the Ethics Committee with the protocol number is mentioned in the informed consent document. The informed consent documents use a unique user identification code for anonymization of personal information and will be signed by both the participants and the researcher that conducts the interview and information session as well as by the principal investigator of the study. A special section of the informed consent form for participants aged under 18 years requires the approval of their participation by their parent/guardian, without which they cannot take part in the study. Information provided in the consent form is described below:

- Participation is strictly voluntary.
- Participants have the right to ask questions about the study procedures before taking part in the study.
- All underlying risks or burdens of their participation would be made clear to participants beforehand.
- Participants will be made aware about what the benefits (scientific, otherwise) will be from this study.
- Participants will be informed specifically about how their data will be collected, handled, and protected during the project’s lifetime as well as whether data will be destroyed or reused (in case there is a possibility to reuse them).
- Participants will have to agree with the further use of their data.
- Participants are informed that they can leave the study at any time or withdraw their data from the study.
- Participants will be informed about the possible commercial exploitation of the research.

### Recruitment of Participants

Estimated enrollment to the NeuroSuitUp pilot study was set for 20 participants (10 patients with SCI and 10 age- and sex-matched healthy participants). The allocation is nonrandomized, nonblind with a parallel assignment intervention model controlled by a cohort of healthy participants and no masking (open label). There are 3 study arms: (1) experimental—complete injury at cervical spine (n=5 participants); (2) experimental—incomplete injury at cervical spine (n=5 participants); and (3) active comparator (n=10 healthy participants).

While the sample size in pilot trials is suggested to not fall within the requirements for power analysis [22], the enrollment for the NeuroSuitUp pilot study was estimated by having in mind the research experience of the research group with patients with SCI and their particularities from previous research projects [23] as well as suggestions from the literature [24,25]. The clinical trial is set to begin in August 2022, while patient enrollment into the clinical trial is estimated to begin in January 2023 and end by December 2023.

The participants will be selected from the SCI and healthy general population in Greece. As described before, the study design allows recruitment of patients at least 14 years of age. The inclusion of the adolescent population in the study was deemed necessary due to the bimodal distribution of SCI demographics [26,27], in which a large portion of the patients’ age at the time of injury ranges from 13-16 to 30 years. SCI is very rare in ages lower than that due to reasons related to both skeletal immaturity and behavioral characteristics of preadolescent children. Recruitment will be facilitated by the Second Neurosurgical Department of Ippokrateio General Hospital of Thessaloniki and the Department of Physiotherapy of International Hellenic University that both support the implementation of pilot trials and the relevant dissemination activities. As such, patients with SCI can be reached through the patients previously or currently treated by these 2 departments and new patients can be prospectively informed about future possibilities in case their condition reaches the chronic phase. Moreover, the collaborating departments will assist in the dissemination of information to the relevant population and the public through their websites and patient organizations, through supporting dissemination events (information days, layouts) and through their participation in scientific publications.

### Experimental Procedures

#### Overview

**Figure 4:**
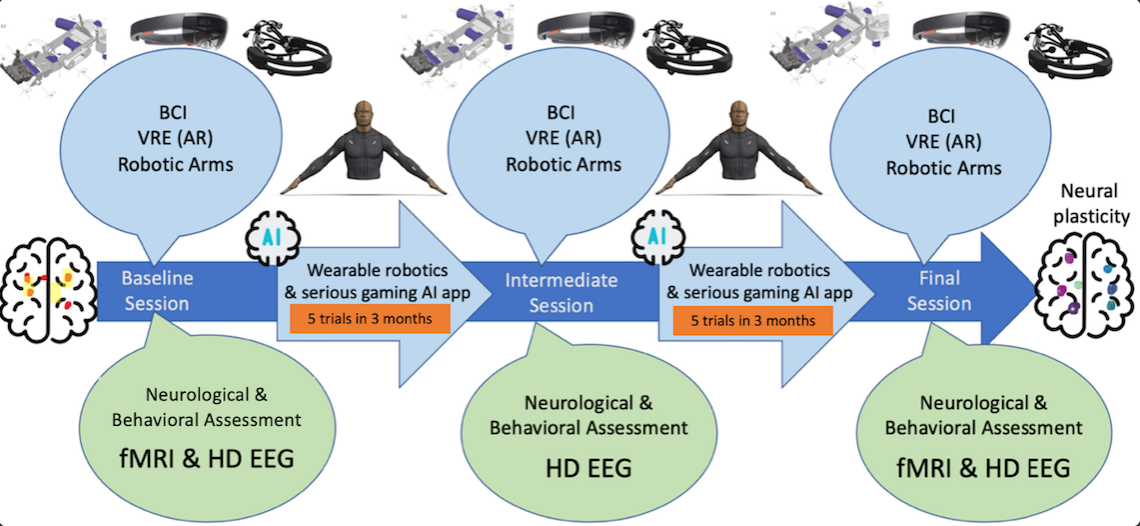
Overview of the experimental procedures, based around 3 phases (initial, intermediate, and final assessments). AI: artificial intelligence; AR: augmented reality; BCI: brain-computer interface; fMRI: functional magnetic resonance imaging; HD EEG: high-density electroencephalography; VRE: virtual reality environment.

The intervention phase will be based on 3 main assessment sessions (initial, intermediate, and final), in which neurological, behavioral, and neurophysiological assessment (EEG) will be performed. Before the initial session, a brain MRI scan of the participants will also be performed. The 3 sessions will span across 6 months for each participant, 3 months between initial and intermediate, and 3 months between intermediate and final. The intervention procedures will consist of the 4 components described earlier. Between the main sessions, the participants will participate in regular training sessions of the BMI (wearable robotics) modality (5 sessions/3 months). The interventions are depicted in Figure 4.

#### Initial Assessment Session

After signing the informed consent form, participants will be screened for inclusion and exclusion criteria and they will be administered the questionnaires presented in the relevant subsection. The participants (and together with their caregivers) will then be taken to the collaborating hospital for a functional MRI of the brain and will return to the Medical Physics and Digital Innovation Laboratory site. Breaks will be provided whenever requested by the participant. On-site, the participants will enter the EEG room to practice the VMI module and will then enter the Thess-AHALL Living Lab [28] room and practice controlling the robotic anthropomorphic limbs as described before. Finally, participants will additionally wear the Microsoft HoloLens 2.0 device and practice controlling an avatar and virtual limbs in an AR environment in multiple movements (multiple degrees of freedom) and functional object movement exercises, with control through mental movement and BCI. A range of wearable sensors for physiological recordings (heart rate, pressure, skin conductance) will also be included.

#### Intermediate and Final Assessment Sessions

The procedures of the intermediate and final assessment sessions are identical to the initial one with the exception of (1) signing an informed consent form, (2) screening for inclusion criteria, and (3) performing a functional brain MRI, all of which will not be repeated.

#### Regular Training Sessions

Participants will enter the Thess-AHALL Living Lab room and practice controlling a wireless wearable BMI (wearable robotics jacket and gloves) with limb position and pressure sensors, EMG, and EMS actuators with haptic feedback that will be used to train residual movements of the upper extremities. They will be asked to complete motor exercises within the dojo-themed SG. The exercises will include finger and hand movements and the reward method will include music and visual stimuli in different difficulty settings. Music in this setting will be used as both a control and a reward. A range of wearable sensors for physiological recordings (heart rate, pressure, skin conductance) will also be included. Breaks will be provided whenever requested by the participant. Finally, the participants will be given the test questionnaires presented in the next subsection.

#### Questionnaires

##### Initial, Intermediate, and Final Assessment Sessions

- International Standards for Neurological Classification of Spinal Cord Injury [1]
- AIS [1]
- Greek translation of the Spinal Cord Independence Measure, version III (g-SCIM-III) [29,30]
- Modified Ashworth Scale [31]
- Beck Depression Inventory (BDI) [32,33]
- Beck Anxiety Inventory (BAI) [34]
- Apathy Evaluation Scale—Clinician version (AES-C) [35]
- Rosenberg Self-Esteem Scale (RSES) [36,37]
- 10-item Kinesthetic and Visual Imagery Questionnaire (KVIQ-10) [38]
- Vividness of Visual Imagery Questionnaire (VVIQ) [39]
- GODSPEED Robotics Questionnaire [40]
- Subjective Mental Effort Questionnaire (SMEQ) [41]
- Locally Experienced Discomfort Questionnaire (LED) [42]

##### Regular Training Sessions

- GODSPEED Robotics Questionnaire [43]
- KVIQ-10
- SMEQ
- LED
- AES-C

### Outcome Measures of the Pilot Study

#### Primary Outcome Measures

##### The BCI Control (Yes/No)

This is defined as the ability of participants to modulate brainwave activity to achieve control of the BCI. BCI control is evaluated as achieved or not (there are cases of BCI illiteracy when the participants cannot modulate their brainwaves to control the BCI). Time frame: after the initial assessment session.

##### The SG Performance (In-Game Scoring System)

This is defined as the ability of participants to control the wearable robotic jacket to complete in-game tasks and collect more points. The points will be gathered by matching the speed and position of the in-game task instructions while receiving assistance from EMS. Time frame: at the intermediate assessment session.

#### Secondary Outcome Measures

##### Initial Functional Improvement

This is defined as daily functionality as measured by the g-SCIM-III. Time frame: at the intermediate assessment session.

##### Intermediate Functional Improvement

This is defined as daily functionality as measured by the g-SCIM-III. Time frame: at the final assessment session.

##### Long-Term Functional Improvement

This is defined as daily functionality as measured by the g-SCIM-III. Time frame: 6 months after the final assessment session.

##### BCI Performance

This pertains to the classification accuracy (percentage of voluntary nonerroneous commands to overall number of detected commands) and bit rate (number of commands per minute): defined as the achieved performance on BCI at the conclusion of BCI sessions for each participant. Measured by classification accuracy (percentage of voluntary non-erroneous commands to overall number of detected commands) and by bit rate (number of commands per minute). Time frame: at the final assessment session.

### Pilot Testing During Development

Before conducting the pilot study with formally enrolled participants in the protocols, we conducted test trials by healthy individuals, who tested the various components for feasibility of the protocol and especially the BMI consisting of wearable robotics jacket and gloves in combination with the SG application component. The BMI component as the most newly developed modality of the designed interventions and procedures was evaluated for easiness of use, for possible discomfort, and for the perception of the robotics game platform by the participants. They performed basic tasks (raising arms consecutively) attempting to match the arm position of the game avatar for 3 minutes. User experience was assessed by the SMEQ [6] and the LED [7] scales. Perception of the robotics game platform was assessed using the Greek version of the Godspeed Robotics Questionnaire (Godspeed-g-1.2). Participants also provided qualitative data in an open format.

### Statistical Analysis

Statistical analysis of the preliminary data as well as of the final pilot study data will be performed using SPSS (version 26.0; IBM, Inc.) and the statistical significance level will be set at .05. Continuous variables are explored for normality by means of the Shapiro-Wilk test and the appropriate descriptive statistics (parametric/non-parametric) will be used accordingly. Normally distributed continuous variables are reported as mean (SD), while nonnormally distributed variables are reported as median and interquartile range (Q1-Q3). Regarding the test trials, possible associations between variables were investigated using the Spearman correlation coefficient.

### Data Availability

Data from the NeuroSuitUp study will be made publicly available after the project’s completion and will be accessible through the institutional project web page under an Attribution-NonCommercial-NoDerivatives 4.0 International license. Data of the preliminary analysis presented in this manuscript can be made available via a request to the authors following a Memorandum of Understanding in the context of Open Research Initiative.

## Results

### Demographics

In total, 10 participants (8 male and 2 female) with a mean age of 31.90 (SD 5.80) years participated in the test trials (conducted during March-May 2022) of the platform consisting of the wearable robotics and the SG application. Analysis of results from the main clinical trial will begin as recruitment progresses and according to completion of the 3 phases by the participant in a rolling fashion. Findings from the complete analysis of results are expected in early 2024.

### Subjective Mental Effort Questionnaire

**Figure 5:**
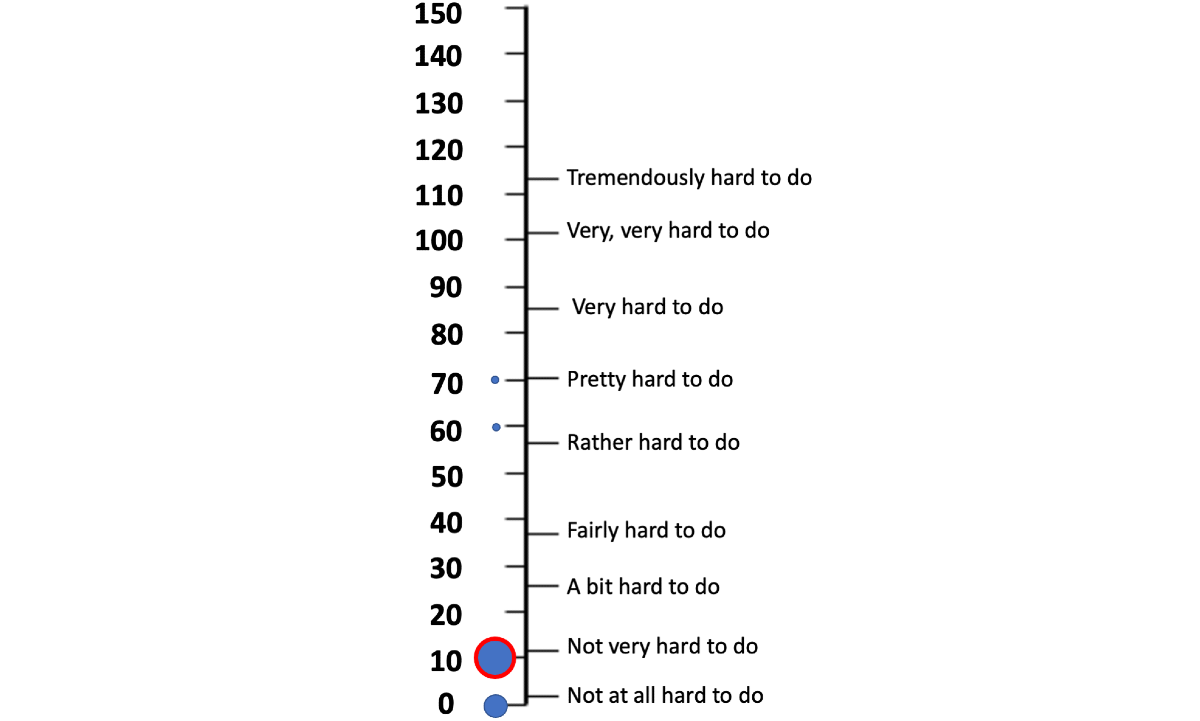
Answers of test trials participants to SMEQ. Size of the circles denotes relative number of answers. Red circle denotes median marking. SMEQ: Subjective Mental Effort Questionnaire.

The participants in the test trials reported that the wearable robotics and SG platform were not very hard to use, as in the SMEQ scale the median rating was 10 (IQ1=5, IQ3=10) (not very hard to do). Most participants (8/10) answered either “not very hard to do” or “not at all hard to do,” while only 2 participants answered either “rather hard to do” or “pretty hard to do,” as seen in Figure 5.

### Locally Experienced Discomfort

**Figure 6:**
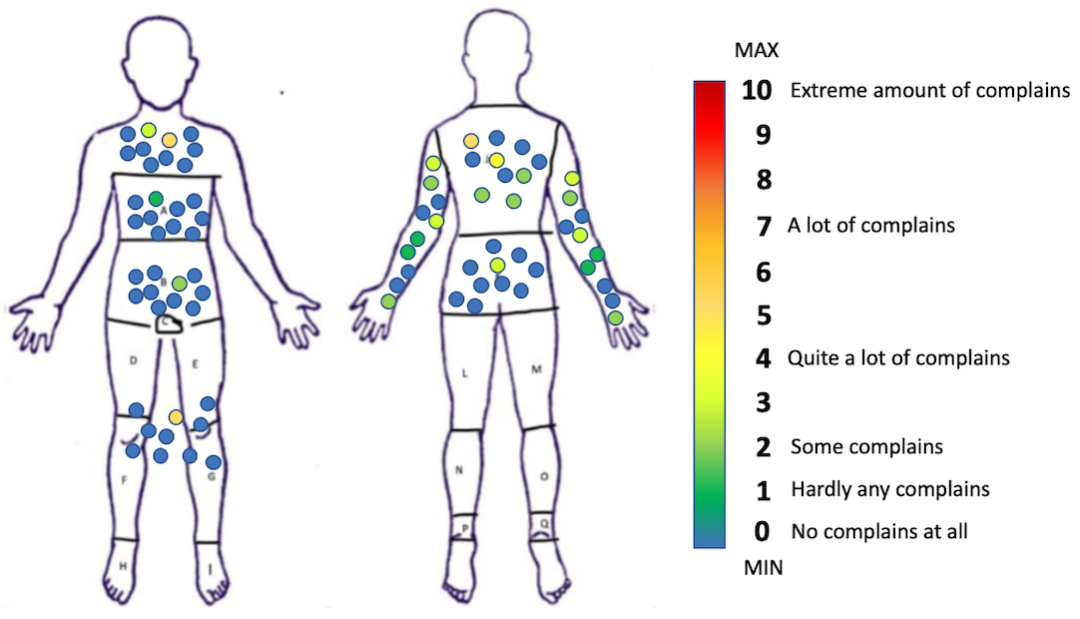
All answers of test trials participants to the Locally Experienced Discomfort Questionnaire according to body area. Color inside the circles corresponds to complain intensity according to colormap.

Very little local discomfort was elicited, as no body area was reported with a median over 1 in the LED scale and no participant utilized a marking of more than 5 in the same scale for any of the body areas. Most complains were reported in the back and the arms, as seen in Figure 6, which depicts all the answers of the participants for the different body parts. Both legs were marked as a single area, and so were the front and back surface of either arm.

### Godspeed Robotics Questionnaire (Godspeed-g-1.2)

**Figure 7:**
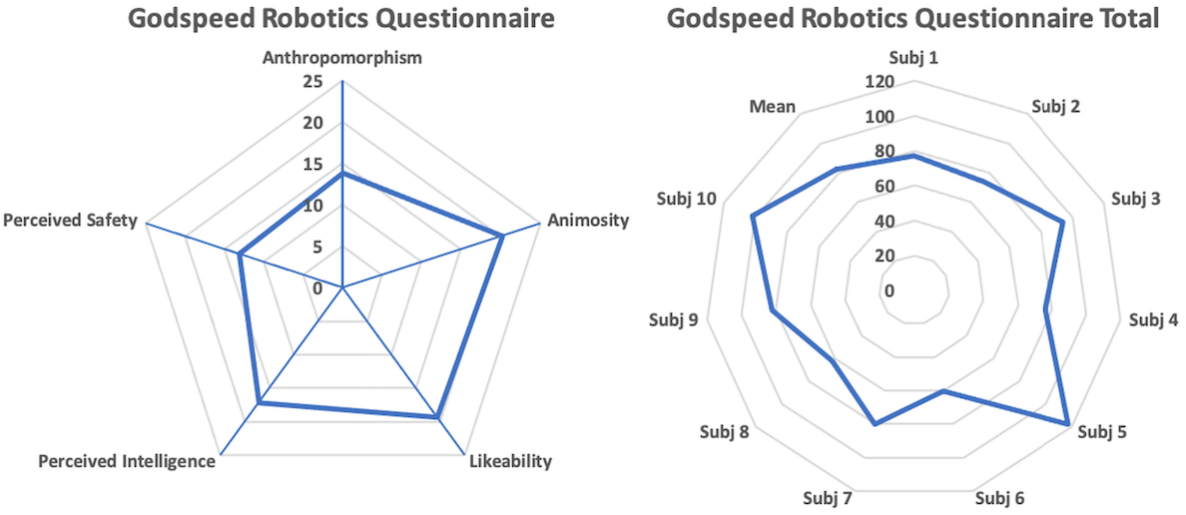
(A) Mean Godspeed robotics questionnaire scores by category. (B) Total Godspeed scores of all participants in the test trials.

The participants in the test trials perceived the robotics game platform positively, as mean total Godspeed score was 82.4 (SD 17.58; maximum 120). Regarding Godspeed subcategories, mean Anthropomorphism was 13.8 (SD 5.05; maximum 25), mean Animosity 20.2 (SD 5.51; maximum 30), mean Likeability 19.3 (SD 4.67; maximum 25), mean Perceived Intelligence 17.1 (SD 5.63; maximum 25), and mean Perceived Safety 13.1 (SD 2.80; maximum 20), as depicted in Figure 7. No correlations were revealed between questionnaires.

## Discussion

### Expected Findings

In summary, according to the preliminary results reported before, the current test version of the platform was assessed as easy to use, causing little to no discomfort and generally viewed as a safe and positive experience by the participants at the test trials. This allowed the research team to proceed with the recruitment for the NeuroSuitUp pilot study, which, as described in the study design, will be a nonrandomized controlled clinical trial, assessing the use of the man-machine interfaces included in the platform by patients with SCI.

Regarding the arm including patients with incomplete injury, we hypothesized mild improvement in functional independence, as spinal cord independence measured is anticipated within main outcomes of the study. This would not only demonstrate the feasibility of the different noninvasive man-machine–based components of the platform as suitable treatment modalities for incomplete SCI at the cervical spine, but also validate previously reported results by patients with lower SCI, where those with dormant neural plasticity can be recruitable even in chronic SCI stages [6]. Such findings, if confirmed, could merit popularization of man-machine–based interventions for the majority of current incomplete SCI population.

Regarding the arm including patients with complete injury, functional improvement, while hypothesized, might not be observed due to the brevity and low intensity of the intervention. Nonetheless, the main outcomes for this arm (as well as the others) are BCI control and performance in the SG-wearable robotics module. Participation may prime such patients to the use of noninvasive interfaces, improve their perception of robotics [3], while also maintaining their motivation and improving their KMI capacity. Improvement in the main and secondary outcomes of the pilot study in this arm may prepare these patients for long-term commitment to relevant interventions. Finally, neurophysiological measurements (EEG, EMG, inertial motion units, and others) from the 3 phases and the training sessions of the study are anticipated to allow us to describe a model of CNS muscle activations and connectivity along injury severity and to further investigate adaptive and maladaptive neural plasticity phenomena.

### Strengths and Limitations of the Study

The advantages provided by the NeuroSuitUp components are the multiple man-machine interfaces that are targeting both CNS and muscle system, the immersiveness of wearables and AR platform, the gamification motivation factor of the SG, and the synergistic nature of the different modalities. Components of the VMI and robotics arms have been previously tested in research projects and proven to be robust and feasible for such a scale of study. The advantages of the wearable components are the multitude of available measurements in real time, as well as the direct control of the intended movement through the intention-EMG-EMS-actuation bridge mediated by the SG training game. In short, the NeuroSuitUp platform offers a noninvasive and holistic solution for interventions in motor disability, such as in chronic SCI, as well as a multibiosignal recording platform to further understand and promote neurophysiological research in such conditions. Furthermore, through the use of the neuropsychological/behavioral battery, our study may enable quantitative data analysis assessing both behavioral characteristics and in-SG performance of the user.

However, there are also limitations that follow the use of noninvasive neural interfacing techniques both in recording, such as EEG and surface EMG (sEMG), and in actuation using EMS. sEMG infers an approximation of activations of underlying muscle groups and may have limited sensitivity in alterations of impacted muscles activation. Similarly, few-channel EEG systems may be appropriate for use in BCI systems but their ability to decode complex motor intention or classify multiple mental commands is also limited. In this case, researchers are trading-off between noninvasiveness and unobtrusiveness from one side and quality and specificity of biosignals from the other, with the optimal choice being subjective to the application and implementation of the techniques. Regarding EMS, the prolonged use of neuromuscular stimulation may produce discomfort and fatigue effects that we are attempting to regulate through duty-cycle stimulation control and through optimization of the motor tasks regarded for training. Regarding the intervention protocol, the total (n=13) number of total sessions (10 assessment and 3 training) may impact adherence and lead to some participants dropping out, especially if no visible neurological improvement is noticeable. We plan to combat this risk by producing an entertaining SG module with many gamification elements to attempt to improve adherence to the protocol.

### Future Work

Initial testing for the NeuroSuitUp wearable components, with 10 participants, provided necessary insight for further improvements, further enabling ease of use, comfort, and functionality. The efforts for the second version of the modality include on-board sensor stabilization for increased freedom of movement, software optimization to decrease any dissociation caused by a delayed response of the system, and modularization to further accommodate possible restriction of the wearer (ie, in wheelchair). We aim to enhance the platform by introducing several device improvements, including full power autonomy to improve unobtrusiveness as well as the design and development of assorted wearable robotics trousers, that will initially aim to gather EMG and magnetic acceleration rotation gravity information, while partially assisting with EMS; body weight support and rigidity will be examined in the future. In that direction, the inclusion of a BCI, in conjunction with the implementation of a hard exoskeleton through the use of lightweight, 3D-printed structures, will allow for the use of more advanced interfacing and control schemes. In particular, the combination of motor imagery potentials from a portable BCI and the preexisting inertial sensors would open the platform to patients that have no residual muscle activation, while the introduction of external pneumatic actuators on the exoskeleton would allow the system to circumvent physiological restrictions of the existing system, specifically muscle fatigue from the continuous use of the EMS. Finally, we aim to broaden the applicability of the platform in the coming months, with a version of the wearable system aiming at patients with stroke-related movement disabilities.

### Conclusions

Chronic SCI is characterized by an often irreversible disability impacting functional independence. Wearable and soft robotics using body-machine and brain-machine interfaces for their control have been increasingly popular in neurological condition rehabilitation research. Immersive serious gaming applications have demonstrated added value in the ability to induce motivation and adherence to rehabilitation regimens. In order for such applications to achieve ethical and end user acceptance for everyday tasks, the use of noninvasive, unobtrusive, safe, and relatively low-cost systems is required. NeuroSuitUp could provide a valuable complementary platform for training in immersive rehabilitation methods to promote dormant neural plasticity. While motor control and assistance are the main focus of the platform, the used biosignals are able to track affect states of users in real time, assessing emotional impact [44], learning gain [45], as well as engagement and motivation to provide rehabilitation experience tailored real-time to user’s interest, attention, and effort.

## Appendix

Multimedia Appendix 1 External peer-review from the European Social Fund - Operational Programme Human Resources Development, Education, and Lifelong Learning 2014-2022 (Greece and European Union).

## Abbreviations

AES-C: Apathy Evaluation Scale—Clinician version
AIS: ASIA Impairment Scale
AR: augmented reality
ASIA: American Spinal Injury Association
BAI: Beck Anxiety Inventory
BCI: brain-computer interface
BDI: Beck Depression Inventory
BMI: body-machine interface
CNS: central nervous system
EEG: electroencephalography
EMG: electromyography
EMS: electrical muscle stimulation
fMRI: functional magnetic resonance imaging
g-SCIM-III: Greek translation of the Spinal Cord Independence Measure, version III
KMI: kinesthetic motor imagery
KVIQ-10: 10-item Kinesthetic and Visual Imagery Questionnaire
LED: Locally Experienced Discomfort Questionnaire (scale)
MRI: magnetic resonance imaging
ROS: robot operating system
RSES: Rosenberg Self-Esteem Scale
SCI: spinal cord injury
sEMG: surface electromyography
SMEQ: Subjective Mental Effort Questionnaire
US NIH/NINDS: US National Institute of Neurological Disorders and Stroke
VMI: visual motor imagery
VRE: virtual reality environment
VVIQ: Vividness of Visual Imagery Questionnaire
