# Supplementary material for: Neurorehabilitation Through Synergistic Man-Machine Interfaces Promoting Dormant Neuroplasticity in Spinal Cord Injury: Protocol for a Nonrandomized Controlled Trial
Source: JMIR Res Protoc. 2022 Sep 13;11(9):e41152. doi: 10.2196/41152 (PMC9516361; doi:10.2196/41152)
Supplement: Multimedia Appendix 1 [file resprot_v11i9e41152_app1.pdf]

ΑΞΙΟΛΟΓΗΣΗ ΕΡΕΥΝΗΤΙΚΗΣ ΠΡΟΤΑΣΗΣ

NeuroSuitUp: Νευροαποκατάσταση μέσω συνεργικών διεπαφών ανθρώπου-μηχανής, προάγοντας την αδρανή νευροπλαστικότητα στην κάκωση νωτιαίου μυελού.

Κωδ. Πρότασης: 100336
Αρ. Αίτησης: 855

Βαθμολογία

Βαθμός 1ης αξιολόγησης: 98,30
Βαθμός 2ης αξιολόγησης: 82,75
ΤΕΛΙΚΟΣ ΒΑΘΜΟΣ: 90,52

1η Αξιολόγηση

Α. Πληρότητα και σαφήνεια του περιεχομένου της πρότασης

Βαθμός: 98/100

Σχόλια/Παρατηρήσεις

Η πρόταση είναι προσεκτικά γραμμένη, αναλυτική και παραθέτει με σαφήνεια, και σε διακριτά μέρη του κειμένου, τις απαντήσεις στα κριτήρια που τίθενται προς αξιολόγηση. Το αντικείμενο της έρευνας περιγράφεται με σαφήνεια, τα ερευνητικά ερωτήματα είναι ξεκάθαρα δοσμένα υπό μορφή λίστας και είναι ιδιαίτερα ενδιαφέροντα, όπως και όλη η πρόταση. Η μεθοδολογία δίνεται ιδιαίτερα αναλυτικά και είναι σχεδιασμένη σε μεγαλύτερο βαθμό από ότι θα απαιτείτο στη φάση της πρότασης. Η συνεισφορά στην επιστημονική γνώση είναι προφανής, όπως και η συνολική χρησιμότητα της πρότασης.

Β. Καινοτομία της ερευνητικής πράξης

Βαθμός: 98/100

Σχόλια/Παρατηρήσεις

Το προτεινόμενο έργο είναι ιδιαίτερα καινοτόμο, τόσο διότι κινείται σε ένα χώρο που από μόνος του αποτελεί καινοτομία, αλλά και διότι η μεθοδολογία εμπεριέχει καινοτόμα στοιχεία, όπως τη χρήση πολλαπλών διεπαφών ανθρώπου μηχανής, την ταυτόχρονη χρήση, τόσο των ρομποτικών όσο και των εικονικών άκρων σε περιβάλλον επαυξημένης πραγματικότητας, αλλά και τα φορετά ρομποτικά συστήματα. Ο συνδυασμός σοβαρού παιγνίου σε συνδυασμό με τη ρομποτική βοηθητική τεχνολογία και η συμβολή της μουσικής και των οπτικών ερεθισμάτων αποτελούν επίσης καινοτόμα στοιχεία.

Γ. Ρεαλιστικότητα του χρονοδιαγράμματος

Βαθμός: 100/100

Σχόλια/Παρατηρήσεις

Το χρονοδιάγραμμα είναι άριστα σχεδιασμένο και παρουσιασμένο. Το διάγραμμα Gantt είναι ολοκληρωμένο, με τα σημεία ορόσημα και τα παραδοτέα να περιγράφονται αναλυτικά. Σε διακριτό τμήμα γίνεται η προσπάθεια

στοιχειοθέτησης της ρεαλιστικότατης την οποία βρίσκω επιτυχημένη.

Δ. Σύνθεση Ερευνητικής Ομάδας και κατανομή ρόλων των μελών της ομάδας

Βαθμός: 98/100

Σχόλια/Παρατηρήσεις

Η σύνθεση της ερευνητικής ομάδας και το προφίλ του ακαδημαϊκού συμβούλου εγγυάται επιτυχές αποτέλεσμα στο έργο. Τα μέλη της είναι από διαφορετικές επιστημονικά πεδία τα οποία καλύπτουν τις ανάγκες του προτεινόμενου έργου.

Βαθμός 1ης αξιολόγησης: 98,3

2η Αξιολόγηση

Α. Πληρότητα και σαφήνεια του περιεχομένου της πρότασης

Βαθμός: 85/100

Σχόλια/Παρατηρήσεις

(α) Η περιγραφή του αντικειμένου της έρευνας και των ερωτημάτων που θέτει είναι σχετικά σαφής (β) η μεθοδολογία υλοποίησης της έρευνας είναι πολύ καλή, (γ) η επάρκεια του εννοιολογικού πλαισίου είναι καλή, (δ) η συνεισφορά στη θεωρητική ή/και εφαρμοσμένη επιστημονική γνώση είναι πολύ μεγάλη εάν τα αποτελέσμτα είναι θετικά

Β. Καινοτομία της ερευνητικής πράξης

Βαθμός: 85/100

Σχόλια/Παρατηρήσεις

Ο βαθμός καινοτομίας της προτεινόμενης Πράξης, ο οποίος, ενδεικτικά και μεταξύ άλλων, μπορεί να τεκμηριώνεται με βάση την εφαρμογή νέων ερευνητικών προσεγγίσεων, μεθοδολογίας ή/και εννοιολογικών πλαισίων είναι μεγάλος

Γ. Ρεαλιστικότητα του χρονοδιαγράμματος

Βαθμός: 80/100

Σχόλια/Παρατηρήσεις

(α) Η προοπτική ολοκλήρωσης της έρευνας στο προβλεπόμενο χρονικό διάστημα που διαρκεί η πράξη είναι καλά στοιχειοθετημένη , (β) οι ανάγκες της προτεινόμενης μεθοδολογίας συνάδουν με το προτεινόμενο χρονοδιάγραμμα.με πολλές δυσκολίες

Δ. Σύνθεση Ερευνητικής Ομάδας και κατανομή ρόλων των μελών της ομάδας

Βαθμός: 80/100

Σχόλια/Παρατηρήσεις

(α) το προφίλ των μελών της ερευνητικής ομάδας βάσει του βιογραφικού τους είναι πολύ καλό!, (β) η ακαδημαϊκή τους εμπειρία μεγάλη (γ) η ποιότητα των δημοσιεύσεων καλή, (δ) η ενδεχόμενη επαγγελματική εμπειρία σε συναφές ερευνητικό πεδίο μεγάλη, (ε) η συνολική επάρκεια της ομάδας να υλοποιήσει την προτεινόμενη έρευνα, (στ) ο τρόπος με τον οποίο έχουν καταμεριστεί οι ρόλοι στο εσωτερικό της ερευνητικής ομάδας σε σχέση με το προφίλ του κάθε μέλους, ικανοποιητική, (ζ) η ικανότητά του Ακαδημαϊκού Συμβούλου να ανταποκριθεί στο ρόλο του μεγάλη

Βαθμός 2ης αξιολόγησης: 82,75

Ευρωπαϊκή Ένωση
Ευρωπαϊκό Κοινωνικό Ταμείο
Επιχειρησιακό Πρόγραμμα
Ανάπτυξη Ανθρώπινου Δυναμικού, Εκπαίδευση και Διά Βίου Μάθηση
Με τη συγχρηματοδότηση της Ελλάδας και της Ευρωπαϊκής Ένωσης
ΕΣΠΑ 2014-2020
ανάπτυξη - εργασία - αλληλεγγύη

EVALUATION OF RESEARCH PROPOSAL

NeuroSuitUp: Neurorehabilitation through synergistic man-machine intefaces promoting dormant neuroplasticity in spinal cord injury

Proposal code: 100336
No. of application: 855

Scoring

1st evaluation mark: 98.30
2nd evaluation mark: 82.75
FINAL MARK: 90.53

1st Evaluation

A. Completeness and clarity of proposal content
Mark: 98/100
Comments/Remarks
The proposal is carefully written, analytical and clearly lays, and in separate text parts, the answers to the criteria that are set for evaluation. The research is described clearly, the research questions are clearly given in list format and are quite interesting, so is the proposal as a whole. The contribution to the scientific knowledge is obvious, so is the overall usefulness of the proposal.

B. Innovation of research act
Mark: 98/100
Comments/Remarks
The proposed project is especially innovative, much why it is on a field that constitutes innovation on its one, but also because the methodology includes innovative elements, like the use of multiple man-machine interfaces, concomitant use of robotic arms and virtual arms in virtual reality environments but also wearable robotic systems. The combination of serious game to assistive robotic technology and the contribution of music and visual feedback constitute innovative elements as well.

C. Timeplan realism
Mark: 100/100
Comments/Remarks
The time plan is excellently designed and presented. The Gantt chart is complete with the milestones and deliverables well described. In a separate section it is attempted to support realism that I find to be successful.

D. Research Team Constitution and team member role distribution
Mark: 98/100
Comments/Remarks
The constitution of the research team and the profile of the primary investigator guarrante a successful outcome to the project. The members come from different scientific fields that cover the necessities of the proposed project.

1st evaluation mark: 98.30

2nd Evaluation

A. Completeness and clarity of proposal content
Mark: 85/100
Comments/Remarks
(a) The description of the proposal is and the research questions are relatively clear, (b) the methodology to complete the research is very good, (c) the adequateness of conceptual framework is good, (d) the contribution to theoretical and/or applied scientific knowledge is very great if the results are positive

B. Innovation of research act
Mark: 85/100
Comments/Remarks
(a) The prospect of completing the research in the foreplaned period of time that the call lasts is documented (b) the needs of the proposed methodology are consistent to the proposed timeplan with a lot of difficulties

C. Timeplan realism
Mark: 80/100
Comments/Remarks
The time plan is excellently designed and presented. The Gantt chart is complete with the milestones and deliverables well described. In a separate section it is attempted to support realism that I find to be successful.

D. Research Team Constitution and team member role distribution
Mark: 80/100
Comments/Remarks
(a) the profile of the research team members based on their resume is very good!, (b) their academic experience is great (c) the quality of their publications is good, (d) the possible occupational experience in a related field is great, (e) the total adequateness of the research team to implement this research, (f) the way the roles have been distributed inside the research team with regards to the profile of each member is satisfactory, (g) the ability of the primary investigator to fulfill their role is great.

2nd evaluation mark: 82.75

European Union
European Social Fund
Operational Programme
Human Resources Development,
Education and Lifelong Learning
Co-financed by Greece and the European Union
ΕΣΠΑ
2014-2020

ΕΛΛΗΝΙΚΗ ΔΗΜΟΚΡΑΤΙΑ
ΥΠΟΥΡΓΕΙΟ ΑΝΑΠΤΥΞΗΣ & ΕΠΕΝΔΥΣΕΩΝ

ΑΝΑΡΤΗΤΕΑ ΣΤΟ ΔΙΑΔΙΚΤΥΟ

ΕΙΔΙΚΗ ΥΠΗΡΕΣΙΑ ΔΙΑΧΕΙΡΙΣΗΣ Ε.Π. "ΑΝΑΠΤΥΞΗ ΑΝΘΡΩΠΙΝΟΥ ΔΥΝΑΜΙΚΟΥ, ΕΚΠΑΙΔΕΥΣΗ & ΔΙΑ ΒΙΟΥ ΜΑΘΗΣΗΣ"
Ταχ. Δ/νση: Κοραή 4
Αθήνα, 105 64
Πληροφορίες: ΙΣΜΗΝΗ ΓΟΥΡΓΙΩΤΟΥ
Τηλ.: 2103278116, 210 5201200
Email: gourgiotou@epeaek.gr

Αθήνα, 19/11/2019
Α.Π.: **5896**

Προς:
ΕΙΔΙΚΟΣ ΛΟΓΑΡΙΑΣΜΟΣ ΚΟΝΔΥΛΙΩΝ ΕΡΕΥΝΑΣ ΑΡΙΣΤΟΤΕΛΕΙΟΥ ΠΑΝΕΠΙΣΤΗΜΙΟΥ ΘΕΣΣΑΛΟΝΙΚΗΣ
Υπ' όψιν Νομίμου Εκπροσώπου
Υπεύθυνου Πράξης ΜΑΓΡΑΣ ΙΩΑΝΝΗΣ

ΘΕΜΑ: Ένταξη της Πράξης «NeuroSuitUp: Νευροαποκατάσταση μέσω συνεργικών διεπαφών ανθρώπου-μηχανής, προάγοντας την αδρανή νευροπλαστικότητα στην κάκωση νωτιαίου μυελού.» με Κωδικό ΟΠΣ 5047840 στο Επιχειρησιακό Πρόγραμμα «Ανάπτυξη Ανθρώπινου Δυναμικού, Εκπαίδευση και Δια Βίου Μάθηση 2014-2020»

ΑΠΟΦΑΣΗ

Η Ειδική Γραμματέας Διαχείρισης Προγραμμάτων Ευρωπαϊκού Ταμείου Περιφερειακής Ανάπτυξης, Ταμείου Συνοχής και Ευρωπαϊκού Κοινωνικού Ταμείου

Έχοντας υπόψη:

1. Το άρθρο 90 του «Κώδικα Νομοθεσίας για την Κυβέρνηση και Κυβερνητικά Όργανα» που κυρώθηκε με το άρθρο πρώτο του Π.Δ. 63/2005 (ΦΕΚ 98/Α/22-4-2005),
2. Το Ν. 4314/2014 για τη διαχείριση, τον έλεγχο και εφαρμογή αναπτυξιακών παρεμβάσεων για την προγραμματική περίοδο 2014 – 2020 (ΦΕΚ 265/Α/23-12-2014), όπως ισχύει,
3. Την Απόφαση της Ευρωπαϊκής Επιτροπής με αριθμό C/2014/10128 final – 17-12-2014 που αφορά στην έγκριση του Ε.Π. «Ανάπτυξη Ανθρώπινου Δυναμικού, Εκπαίδευση και Δια Βίου Μάθηση 2014-2020» (Κωδικός CCI2014GR05ΘOP001), όπως ισχύει,
4. Τους Κανονισμούς 1303/2013 και 1304/2013 του Ευρωπαϊκού Κοινοβουλίου και του Συμβουλίου, όπως ισχύουν,
5. Την Υπουργική Απόφαση με αριθ. 53684/ΕΥΘΥ460/18-05-2015 (ΦΕΚ 948/Β/27-05-2015) με την οποία συστάθηκε η Ειδική Υπηρεσία Διαχείρισης του ΕΠ ΑΝΑΔ ΕΔΒΜ,
6. Το Π.Δ. 81/2019 (ΦΕΚ 119/Α/8-07-2019) «Σύσταση, συγχώνευση, μετονομασία και κατάργηση Υπουργείων και καθορισμός των αρμοδιοτήτων τους - Μεταφορά υπηρεσιών και αρμοδιοτήτων μεταξύ Υπουργείων.»,
7. Το Π.Δ. 83/2019 (ΦΕΚ 121/Α/9-07-2019) «Διορισμός Αντιπροέδρου της Κυβέρνησης, Υπουργών, Αναπληρωτών Υπουργών και Υφυπουργών.»,
8. Το Π.Δ. 84/2019 (ΦΕΚ 123/Α/17-07-2019) «Σύσταση και κατάργηση Γενικών Γραμματειών και Ειδικών Γραμματειών/Ενιαίων Διοικητικών Τομέων Υπουργείων.»,
9. Τη με αρ. πρωτ. 80261/01-08-2019 (ΦΕΚ 511/τ.Υ.Ο.Δ.Δ./02-08-2019) Απόφαση που αφορά στο «Διορισμό μετακλητής Ειδικής Γραμματέως Διαρθρωτικών Προγραμμάτων του Υπουργείου Ανάπτυξης και Επενδύσεων",
10. Το Ν. 4622/2019 (ΦΕΚ 133/Α/07-08-2019) «Επιτελικό Κράτος: οργάνωση, λειτουργία και διαφάνεια της Κυβέρνησης, των κυβερνητικών οργάνων και της κεντρικής δημόσιας διοίκησης»,
11. Την από 01-07-2015 απόφαση της Επιτροπής Παρακολούθησης του Ε.Π. «Ανάπτυξη Ανθρώπινου Δυναμικού, Εκπαίδευση και Δια

Βίου Μάθηση», όπως ισχύει, με την οποία εγκρίθηκε η μεθοδολογία, η διαδικασία και το περιεχόμενο της εξειδίκευσης του ΕΠ, ο προγραμματισμός των προσκλήσεων και των εντάξεων και οι τεθέντες κατ' έτος στόχοι, καθώς και η μεθοδολογία και τα κριτήρια επιλογής των πράξεων των Αξόνων Προτεραιότητας, όπως περιγράφονται και εξειδικεύονται στο συνημμένο στην πρόσκληση έγγραφο,
12. Τη με αρ. πρωτ. 137675/ΕΥΘΥ/1016/19-12-2018(ΦΕΚ 5968/Β/31-12-2018) Υπουργική Απόφαση με θέμα « Αντικατάσταση της υπ' αριθ. 110427/ΕΥΘΥ/1020/20.10.2016 (ΦΕΚ Β΄3521) υπουργικής απόφασης με τίτλο «Τροποποίηση και αντικατάσταση της υπ' αριθ. 81986/ΕΥΘΥ712/31.07.2015 (ΦΕΚ Β΄1822) υπουργικής απόφασης "Εθνικοί κανόνες επιλεξιμότητας δαπανών για τα προγράμματα του ΕΣΠΑ 2014 – 2020 - Έλεγχοι νομιμότητας δημοσίων συμβάσεων συγχρηματοδοτούμενων πράξεων ΕΣΠΑ 2014-2020 από Αρχές Διαχείρισης και Ενδιάμεσους Φορείς – Διαδικασία ενστάσεων επί των αποτελεσμάτων αξιολόγησης πράξεων»,
13.Το Ν. 4485/2017 (ΦΕΚ 114 Α΄) με θέμα: «Οργάνωση και λειτουργία της ανώτατης εκπαίδευσης, ρυθμίσεις για την έρευνα και άλλες διατάξεις», όπως ισχύει,
14. Το Ν.4386/2016 για τις «Ρυθμίσεις για την έρευνα και άλλες διατάξεις», όπως ισχύει,
15.Τη με αρ. πρωτ. 3605/04-07-2019 Πρόσκληση με τίτλο «Υποστήριξη ερευνητών με έμφαση στους νέους ερευνητές-κύκλος Β'» (Κωδ. ΕΔΒΜ103), για την υποβολή προτάσεων στο Ε.Π. "Ανάπτυξη Ανθρώπινου Δυναμικού, Εκπαίδευση και Δια Βίου Μάθηση"
16. Το με ID 75037/31-07-2019 και ώρα 16:16 μ.μ. ΤΔΠ του Δικαιούχου «ΕΙΔΙΚΟΣ ΛΟΓΑΡΙΑΣΜΟΣ ΚΟΝΔΥΛΙΩΝ ΕΡΕΥΝΑΣ ΑΡΙΣΤΟΤΕΛΕΙΟΥ ΠΑΝΕΠΙΣΤΗΜΙΟΥ ΘΕΣΣΑΛΟΝΙΚΗΣ», προς την ΕΥΔ για την ένταξη της πράξης στο Ε.Π. «Ανάπτυξη Ανθρώπινου Δυναμικού, Εκπαίδευση και Δια Βίου Μάθηση»,
17. Το αποτέλεσμα της αξιολόγησης, όπως αυτό καταγράφεται στα έγγραφα τεκμηρίωσης της θετικής αξιολόγησης της πρότασης και ειδικότερα στο Φύλλο αξιολόγησης, το οποίο αποτυπώνεται στο ΟΠΣ –ΕΣΠΑ,
18. Τη με αρ. Υ.Σ.684/13-11-2019 θετική εισήγηση της Προϊσταμένης της ΕΥΔ ΕΠ ΑΝΑΔ ΕΔΒΜ.

Αποφασίζει

την ένταξη της Πράξης «NeuroSuitUp: Νευροαποκατάσταση μέσω συνεργικών διεπαφών ανθρώπου-μηχανής, προάγοντας την αδρανή νευροπλαστικότητα στην κάκωση νωτιαίου μυελού.» στον Άξονα Προτεραιότητας «ΒΕΛΤΙΩΣΗ ΤΗΣ ΠΟΙΟΤΗΤΑΣ ΚΑΙ ΑΠΟΤΕΛΕΣΜΑΤΙΚΟΤΗΤΑΣ ΤΟΥ ΕΚΠΑΙΔΕΥΤΙΚΟΥ ΣΥΣΤΗΜΑΤΟΣ » του Ε.Π. «Ανάπτυξη Ανθρώπινου Δυναμικού, Εκπαίδευση και Δια Βίου Μάθηση».

Η πράξη συγχρηματοδοτείται από το Ευρωπαϊκό Κοινωνικό Ταμείο (ΕΚΤ).

Α. ΣΤΟΙΧΕΙΑ ΠΡΑΞΗΣ

**1.Κωδικός Πράξης/MIS (ΟΠΣ):**	**5047840**
**2.Δικαιούχος:**	**ΕΙΔΙΚΟΣ ΛΟΓΑΡΙΑΣΜΟΣ ΚΟΝΔΥΛΙΩΝ ΕΡΕΥΝΑΣ ΑΡΙΣΤΟΤΕΛΕΙΟΥ ΠΑΝΕΠΙΣΤΗΜΙΟΥ ΘΕΣΣΑΛΟΝΙΚΗΣ**
**3.Κωδικός Δικαιούχου:**	**1020216**

4.Φυσικό αντικείμενο της πράξης:

Αντικείμενο της δράσης είναι η υλοποίηση της ερευνητικής πρότασης "NeuroSuitUp: Νευροαποκατάσταση μέσω συνεργικών διεπαφών ανθρώπου-μηχανής, προάγοντας την αδρανή νευροπλαστικότητα στην κάκωση νωτιαίου μυελού.". Στόχος της παρέμβασης είναι η ενίσχυση του ακαδημαϊκού βιογραφικού των ερευνητών και των ερευνητικών δεξιοτήτων τους, προκειμένου να βελτιωθούν οι προοπτικές ακαδημαϊκής / ερευνητικής καριέρας τους.

5. Παραδοτέα πράξης:

- Εξαμηνιαίες εκθέσεις προόδου
- 1 άρθρο σε διεθνές συνέδριο
- 1 δημοσίευση σε διεθνές επιστημονικό περιοδικό
- Απολογιστική έκθεση αποτελεσμάτων έρευνας

6. ΔΕΙΚΤΕΣ ΕΚΡΟΩΝ				
**ΚΩΔΙΚΟΣ ΔΕΙΚΤΗ**	**ΟΝΟΜΑΣΙΑ ΔΕΙΚΤΗ**	**ΜΟΝΑΔΑ ΜΕΤΡΗΣΗΣ**	**ΚΑΤΗΓΟΡΙΑ ΠΕΡΙΦΕΡΕΙΑΣ**	**ΤΙΜΗ ΣΤΟΧΟΣ**
T4958	Αριθμός ωφελουμένων από δράσεις ενίσχυσης της έρευνας στην τριτοβάθμια εκπαίδευση	Αριθμός	Λιγότερο ανεπτυγμένες περιφέρειες	2,00

ΧΡΟΝΟΔΙΑΓΡΑΜΜΑ ΥΛΟΠΟΙΗΣΗΣ
**7.** Η ημερομηνία έναρξης της Πράξης ορίζεται η **26/11/2019**.
**8.** Η ημερομηνία λήξης της Πράξης ορίζεται η**25/09/2021**.
**10.** Η ανάληψη της νομικής δέσμευσης του πρώτου κύριου υποέργου πραγματοποιείται έως **23/12/2019**.

ΚΑΤΑΝΟΜΗ ΔΗΜΟΣΙΑΣ ΔΑΠΑΝΗΣ ΠΡΑΞΗΣ ΑΝΑ ΚΑΤΗΓΟΡΙΑ ΔΑΠΑΝΗΣ			
ΚΩΔΙΚΟΣ ΚΑΤΗΓΟΡΙΩΝ ΔΑΠΑΝΗΣ		ΣΥΝΟΛΙΚΗ ΔΗΜΟΣΙΑ ΔΑΠΑΝΗ	ΕΠΙΛΕΞΙΜΗ ΔΗΜΟΣΙΑ ΔΑΠΑΝΗ
**Α. ΔΑΠΑΝΕΣ ΒΑΣΕΙ ΠΑΡΑΣΤΑΤΙΚΩΝ**			
Α.1. Άμεσες δαπάνες	i. Ποσό χωρίς ΦΠΑ	46.000,00	46.000,00
	ii. ΦΠΑ	0,00	0,00
**ΣΥΝΟΛΟ ΔΑΠΑΝΩΝ ΜΕ ΠΑΡΑΣΤΑΤΙΚΑ**		46.000,00	46.000,00
**Β. ΔΑΠΑΝΕΣ ΒΑΣΕΙ ΑΠΛΟΠΟΙΗΜΕΝΟΥ ΚΟΣΤΟΥΣ**			
Β.4.1. ΕΜΜΕΣΕΣ ΔΑΠΑΝΕΣ βάσει ποσοστού (%) επί των επιλέξιμων άμεσων δαπανών προσωπικού		46,00	46,00
**ΣΥΝΟΛΟ ΔΑΠΑΝΩΝ ΒΑΣΕΙ ΑΠΛΟΠΟΙΗΜΕΝΟΥ ΚΟΣΤΟΥΣ**		46,00	46,00
**ΣΥΝΟΛΑ**		46.046,00	46.046,00

**ΣΥΝΟΛΙΚΗ ΔΗΜΟΣΙΑ ΔΑΠΑΝΗ**	46.046,00
**ΙΔΙΩΤΙΚΗ ΣΥΜΜΕΤΟΧΗ**	0,00
**ΜΗ ΕΝΙΣΧΥΟΜΕΝΟΣ ΠΡΟΫΠΟΛΟΓΙΣΜΟΣ**	0,00
**ΣΥΝΟΛΙΚΟ ΚΟΣΤΟΣ ΠΡΑΞΗΣ**	46.046,00

11. Η επιλέξιμη δημόσια δαπάνη για τον υπολογισμό της στήριξης της Ένωσης ανέρχεται σε **46.046,00** €

13. Για την Πράξη, οι εφαρμοζόμενες επιλογές απλοποιημένου κόστους είναι οι εξής:

ΔΑΠΑΝΕΣ ΒΑΣΕΙ ΑΠΛΟΠΟΙΗΜΕΝΟΥ ΚΟΣΤΟΥΣ	ΠΑΡΑΜΕΤΡΟΙ ΕΦΑΡΜΟΓΗΣ	ΠΕΔΙΟ ΕΦΑΡΜΟΓΗΣ

Β.4.1 ΕΜΜΕΣΕΣ ΔΑΠΑΝΕΣ βάσει ποσοστού (%) επί των επιλέξιμων άμεσων δαπανών προσωπικού (<= 15%)	0.1	%	

Οι έμμεσες δαπάνες της πράξης υπολογίζονται κατ' αποκοπή με την εφαρμογή του ποσοστού 0,1% επί των επιλέξιμων άμεσων δαπανών προσωπικού.

Β. ΣΤΟΙΧΕΙΑ ΕΓΓΡΑΦΗΣΠΡΑΞΗΣ ΣΤΟ ΠΡΟΓΡΑΜΜΑ ΔΗΜΟΣΙΩΝ ΕΠΕΝΔΥΣΕΩΝ

14. ***Η δημόσια δαπάνη της πράξης*** που προτείνεται για εγγραφή στο Πρόγραμμα Δημοσίων Επενδύσεων ***ανέρχεται σε*** **46.046,00** €

Ηδημόσια δαπάνη της πράξης επιμερίζεται ως ακολούθως:

ΣΥΛΛΟΓΙΚΕΣ ΑΠΟΦΑΣΕΙΣ ΠΟΥ ΧΡΗΜΑΤΟΔΟΤΟΥΝ/ΧΡΗΜΑΤΟΔΟΤΗΣΑΝ ΤΗΝ ΠΡΑΞΗ					
**ΚΩΔ. ΣΑ**	**ΚΩΔ. ΠΡΑΞΗΣΣΑ (ΚΩΔΙΚΟΣ ΕΝΑΡΙΘΜΟΥ)***	**ΠΡΟΤΑΣΗ ΕΓΓΡΑΦΗΣ**	**ΕΝΕΡΓΟΣ ΕΝΑΡΙΘΜΟΣ** (που συνεχίζει να πληρώνει την πράξη)	**Π/Υ ΥΠΟΕΡΓΩΝ ΠΡΟΠΑΡΑΣΚΕΥΑΣΤΙΚΩΝ ΕΝΕΡΓΙΩΝ**	**ΣΥΝΟΛΟ Π/Υ**
Ε3451	2019ΣΕ34510077	Νέο Εργο ΠΔΕ	ΝΑΙ	0,00	46.046,00

* Ο κωδικός εναρίθμου τίθεται σε ισχύ μετά την έγκριση της ΣΑ από τον Υπουργό.

15. Το επιλέξιμο ποσό δημόσιας δαπάνης, η οποία προτείνεται για εγγραφή στο ΠΔΕ ανέρχεται σε **46.046,00** €.

Γ. ΟΡΟΙ ΧΡΗΜΑΤΟΔΟΤΗΣΗΣ

Ο δικαιούχος υποχρεούται να τηρήσει τους όρους της απόφασης ένταξης και να υλοποιήσει την πράξη, σύμφωνα με τους όρους και τον χρονικό προγραμματισμό αυτής, καθώς και να τηρήσει τις υποχρεώσεις που παρατίθενται στο συνημμένο Παράρτημα Ι, το οποίο αποτελεί αναπόσπαστο μέρος της απόφασης ένταξης.

Σε περίπτωση που η υλοποίηση της πράξης αποκλίνει από τους όρους της απόφασης ένταξης, η ΔΑ επανεξετάζει την πράξη και μπορεί να προβεί στην ανάκληση της απόφασης ένταξής της.

Η Ειδική Γραμματέας Διαχείρισης Προγραμμάτων Ευρωπαϊκού Ταμείου Περιφερειακής Ανάπτυξης, Ταμείου Συνοχής και Ευρωπαϊκού Κοινωνικού Ταμείου

Νίκη Δανδόλου

Συνημμένα:

Παράρτημα Ι : ΥΠΟΧΡΕΩΣΕΙΣ ΔΙΚΑΙΟΥΧΩΝ, το οποίο αποτελεί αναπόσπαστο μέρος της Απόφασης Ένταξης

Κοινοποίηση:

1.Ειδική Υπηρεσία Θεσμικής Υποστήριξης, Νίκης 10, 105 63 Αθήνα
2. ΕΠΙΤΕΛΙΚΗ ΔΟΜΗ ΕΣΠΑ ΥΠΟΥΡΓΕΙΟΥ ΠΑΙΔΕΙΑΣ & ΘΡΗΣΚΕΥΜΑΤΩΝ, ΤΟΜΕΑΣ ΠΑΙΔΕΙΑΣ

<u>Εσωτερική διανομή:</u>

1. Γραφείο Υφυπουργού Ανάπτυξης και Επενδύσεων κ. Γ. Τσακίρη
2. Γραφείο Ειδικής Γραμματέως Διαχείρισης Προγραμμάτων Ευρωπαϊκού Ταμείου Περιφερειακής Ανάπτυξης, Ταμείου Συνοχής και Ευρωπαϊκού Κοινωνικού Ταμείου
3. Γραφείο Προϊσταμένης της ΕΥΔ του ΕΠ ΑΝΑΔ ΕΔΒΜ
4. Γραφείο Προϊσταμένου Υποδιεύθυνσης Τομέα Εκπαίδευσης
5. Μονάδα Β 2.2.

—

ΠΑΡΑΡΤΗΜΑ 1: ΥΠΟΧΡΕΩΣΕΙΣ ΔΙΚΑΙΟΥΧΩΝ

Ο δικαιούχος της πράξης «NeuroSuitUp: Νευροαποκατάσταση μέσω συνεργικών διεπαφών ανθρώπου-μηχανής, προάγοντας την αδρανή νευροπλαστικότητα στην κάκωση νωτιαίου μυελού.» αναλαμβάνει να τηρήσει τις παρακάτω υποχρεώσεις :

1. ΤΗΡΗΣΗ ΚΟΙΝΟΤΙΚΩΝ ΚΑΙ ΕΘΝΙΚΩΝ ΚΑΝΟΝΩΝ

(i) Να τηρεί την Κοινοτική και Εθνική Νομοθεσίακατά την εκτέλεση της πράξης και ιδίως όσον αφορά τις δημόσιες συμβάσεις, τη αειφόρο ανάπτυξη, τις κρατικές ενισχύσεις, την ισότητα μεταξύ ανδρώνκαι γυναικών, τη μη διάκριση και την προσβασιμότητα Ατόμων με Αναπηρίες.

2. ΥΛΟΠΟΙΗΣΗ ΠΡΑΞΗΣ

(i) Να τηρεί τα χρονοδιαγράμματα υλοποίησης της πράξης και των επί μέρους υποέργων και ιδίως τα χρονοδιαγράμματα ανάθεσης και ολοκλήρωσης των υποέργων προπαρασκευαστικών ενεργειών και ανάθεσης και εκτέλεσης των κύριων υποέργων της πράξης, όπως αυτά αποτυπώνονται στην απόφαση ένταξης της πράξης.

Τυχόν υπερβάσεις του χρονοδιαγράμματος υλοποίησης των υποέργων προπαρασκευαστικών ενεργειών, όπως αυτό προσδιορίζεται στην απόφαση ένταξης τηςπράξης, επιφέρει την αυτοδίκαιη ανάκληση της απόφασης ένταξης της πράξης (δηλαδή η πράξη απεντάσσεται από το ΕΠ).

Στις περιπτώσεις πράξεων με υποέργα προπαρασκευαστικών υποέργων η εγγραφή του προϋπολογισμού τους γίνεται σε δύο φάσεις. Στην πρώτη φάση εγγράφεται στο ΠΔΕ ο προϋπολογισμός των προπαρασκευαστικών ενεργειών. Εφόσον, τα υποέργα των προπαρασκευαστικών ενεργειών ολοκληρώνονται εντός του χρονοδιαγράμματος, ο προϋπολογισμός της πράξηςπου αντιστοιχεί στα κύρια υποέργα εγγράφεται στο ΠΔΕ, μετά από σχετικό αίτημα της ΔΑ ή του ΕΦ προς τη ΔΔΕ. Συνεπώς,

- Η ανάληψη της κύριας νομικής δέσμευσης δεν μπορεί να υπερβεί την ημερομηνία ανάληψης της πρώτης νομικής δέσμευσης του κύριου υποέργου που ορίζεται στο σημείο10 της Απόφασης Ένταξης. Σε περίπτωση υπέρβασης αυτού του χρονικού ορίου η Απόφαση Ένταξης θα ανακληθεί μετά από προειδοποιητική επιστολή της ΔΑ και την άπρακτη παρέλευση τριών μηνών. Η παραπάνω προθεσμία ισχύει με την επιφύλαξη δικαστικών ή διοικητικών αποφάσεων που αναστέλλουν την υλοποίηση της πράξης ή λόγω ανωτέρας βίας.

Υπερβάσεις του χρονοδιαγράμματος υλοποίησης των κύριων υποέργων δύνανται να επιφέρουν την επιτήρηση του δικαιούχου από τη ΔΑ και την επιβολή στο δικαιούχο λήψης διορθωτικών μέτρων εντός συγκεκριμένων προθεσμιών, αλλά και την ανάκληση της απόφασης ένταξης της πράξης.

(ii) Να διασφαλίζειτο λειτουργικό αποτέλεσμα της πράξης, λαμβάνοντας όλα τα απαραίτητα μέτρα για το σκοπό αυτό, με βάση το κανονιστικό πλαίσιο του φορέα λειτουργίας και συντήρησης της πράξης και των αντίστοιχων αρμοδιοτήτων του, στην περίπτωση που ο φορέας λειτουργίας και συντήρησης της πράξης δεν ταυτίζεται με το δικαιούχο αυτής.

(iii) Να λαμβάνει έγκριση από την Ειδική Υπηρεσία Διαχείρισης του Ε.Π (ή εναλλακτικά τον ΕΦ) γιατις διαδικασίες της διακήρυξης, ανάθεσης και τροποποίησης δημοσίων συμβάσεων. Στις περιπτώσεις πράξεων που εκτελούνταιμε ίδια μέσα, ο δικαιούχος υποχρεούται να υποβάλλει σχέδιο απόφασης για την εκτέλεση του έργου με ίδια μέσα πριν την υπογραφή του, και να υποβάλλει αίτημα εξέτασης για τροποποιήσεις αυτής. Στις περιπτώσεις αρχαιολογικών έργων, ο δικαιούχος κοινοποιεί την απόφαση αυτεπιστασίας.

(iv) Να ενημερώνει έγκαιρα την Ειδική Υπηρεσία Διαχείρισης του Ε.Π (ή εναλλακτικά τον ΕΦ) σχετικά με την εξέλιξη της πράξης, ιδιαίτερα σε ότι αφορά τις προπαρασκευαστικές ενέργειες για την υλοποίησή της και να αποστέλλει όλα ταςχετικά έγγραφα που αφορούν στη φυσική και οικονομική υλοποίηση της πράξης έως και την ολοκλήρωσή της, σύμφωνα με τις διαδικασίες του συστήματοςδιαχείρισης και ελέγχου.

(v) Να πραγματοποιεί όλες τις απαραίτητες ενέργειες, για την ενημέρωση του Ολοκληρωμένου Πληροφοριακού Συστήματος ΟΠΣ – ΕΣΠΑ με τα δεδομένα και έγγραφα της πράξης που υλοποιεί και ειδικότερα τα δεδομένα και έγγραφα προγραμματισμού και υλοποίησης που απαιτούνται για τη χρηματοοικονομική διαχείριση, την παρακολούθηση του φυσικού και οικονομικού αντικειμένου και των δεικτών, τις επαληθεύσεις, τους ελέγχους, την αξιολόγηση πράξεων και γενικότερα τη διαδρομή ελέγχου της πράξης.

(vi) Να διασφαλίζει την ακρίβεια, την ποιότητα και πληρότητα των στοιχείων που υποβάλλει στο ΟΠΣ - ΕΣΠΑ, σύμφωνα με το χρονικό πλαίσιο που προβλέπεταιστις σχετικές διατάξεις και να πραγματοποιεί διασύνδεση των Πληροφοριακών Συστημάτων του μετο ΟΠΣ – ΕΣΠΑ για την αυτόματη υποβολή στοιχείων, εφόσον απαιτείται.

(vii) Να εφαρμόζει το Ενιαίο Σύστημα Παρακολούθησης Δεικτών ΕΣΠΑ, ανάλογα με το επίπεδο εμπλοκής του στην παρακολούθηση των δεικτών της συγχρηματοδοτούμενης πράξης.

(viii) Για πράξεις ΕΚΤ, ο δικαιούχος υποχρεούται να έχει εγκατεστημένο σύστημα (είτε τουφορέα του είτε να έχει πρόσβαση σε άλλο τοπικό ή κεντρικό σύστημα) συλλογής, επεξεργασίας, αποθήκευσης και μεταβίβασης δεδομένων συμμετεχόντων (microdata), όπου απαιτείται, προκειμένου αφενός να διασφαλίζεται η ακρίβεια και επάρκεια των δεδομένων των συμμετεχόντων και αφετέρου η έγκαιρη ενημέρωση του ΟΠΣ – ΕΣΠΑ με τα εν λόγω στοιχεία.

Οιδικαιούχοι έχουν την ευθύνη για την έγκυρη και έγκαιρη συλλογή, επεξεργασία και αποθήκευση των δεικτών και των

δεδομένων μεμονωμένων συμμετεχόντων (microdata) και στιςπεριπτώσεις που η συλλογή τους διενεργείται από τους φορείς υλοποίησης ή παρόχους των πράξεων (π.χ. Κέντρα Επαγγελματικής Κατάρτισης, Δομές φροντίδας παιδιών κα).

3. ΧΡΗΜΑΤΟΔΟΤΗΣΗ ΠΡΑΞΗΣ

(i) Να λειτουργεί μηχανισμό πιστοποίησης εκτέλεσης της πράξης, ο οποίος θα εξασφαλίζει τον αποτελεσματικό έλεγχο τηςποιότητας και ποσότητας των υλικών, των υπηρεσιών και του τελικού παραδοτέου αποτελέσματος, καθώς και να εφαρμόζει εσωτερικές διαδικασίες ελέγχου των πληρωμών, ο οποίος θα εξασφαλίζει τη νομιμότητα και κανονικότητά τους.

(ii) Να τηρεί ξεχωριστή λογιστική μερίδα για τηνπράξη, στην οποία θα καταχωρούνται όλες οι δαπάνες που αντιστοιχούν πλήρως προς τις δαπάνες που δηλώνονται στην Ειδική Υπηρεσία Διαχείρισης του Ε.Π ή στον Ενδιάμεσο Φορέα, μέσω των Δελτίων Δήλωσης Δαπανών.

(iii) Να υποβάλλει (εφόσον απαιτείται από τη φύση του έργου) στην Ειδική Υπηρεσία Διαχείρισης του Ε.Π. (ή εναλλακτικά στον ΕΦ) και στην Αρχή Πιστοποίησης, μετά την ολοκλήρωση της πράξης

α) στοιχεία για τους δημιουργούμενους τόκους από τη χρηματοοικονομική διαχείριση των διατιθέμενων πόρων.

β) επικαιροποιημένη χρηματοοικονομική ανάλυση για τον προσδιορισμό των καθαρών εσόδων για τα έργα που παράγουν έσοδα, εφόσον για τον προσδιορισμό των καθαρών εσόδων εφαρμόζεται η εν λόγω μέθοδος.

Στην περίπτωση πράξης / έργου όπου ο υπολογισμός των καθαρών εσόδων του βασίζεται στη μέθοδο του κατ' αποκοπή ποσοστό (flat rate) δεν απαιτείται να γίνει κάποια προσαρμογή στο ποσοστό χρηματοδότησης της πράξης στην τελική αίτηση πληρωμής που υποβάλλει ο δικαιούχος.

Στην περίπτωση πράξης / έργου που δεν είναι αντικειμενικά δυνατή η εκ τωνπροτέρων εκτίμηση των εσόδων, ο δικαιούχος υποχρεούται να υποβάλλει ετησίως στοιχεία για τα καθαρά έσοδα της πράξης για περίοδο τριών ετών από την ολοκλήρωσή της έως την προθεσμία για την υποβολή της έκθεσης ολοκλήρωσης του ΕΠ, αναλόγως με το ποια χρονική στιγμή προηγείται.

Στην περίπτωση πράξης, η οποία παράγει άμεσα έσοδα μόνο κατά την υλοποίησή της, τα οποία ωστόσο δεν ελήφθησαν υπόψη κατά το χρόνο έγκρισης της πράξης, η επιλέξιμη δαπάνη της πράξης μειώνεται κατά τα καθαρά έσοδα που παρήχθησαν άμεσα κατά τη διάρκεια εκτέλεσής της, το αργότερο κατά την αίτηση τελικής πληρωμής που υποβάλλει ο δικαιούχος.

4. ΕΠΙΣΚΕΨΕΙΣ – ΕΠΑΛΗΘΕΥΣΕΙΣ – ΕΛΕΓΧΟΙ

(i) Να θέτει στη διάθεση, εφόσον ζητηθούν, καθ' όλη τη διάρκεια εκτέλεσης της πράξης και για όσο χρόνο ο δικαιούχος υποχρεούται για την τήρησή τους, όλα τα έγγραφα, δικαιολογητικά και στοιχεία της πράξης, στην Ειδική Υπηρεσία Διαχείρισης του Ε.Π., Αρχή Πιστοποίησης, Αρχή Ελέγχου, Επιτροπή Παρακολούθησης και σε όλα τα ελεγκτικά όργανα της Ελλάδας και της Ευρωπαϊκής Ένωσης.

(ii) Να αποδέχοται επιτόπιους ελέγχους από όλα τα αρμόδια εθνικά και ευρωπαϊκά ελεγκτικά όργανα, τόσο στην έδρα τους, όσο και στους χώρους υλοποίησης της πράξης, και να διευκολύνουν τον έλεγχο προσκομίζοντας οποιοδήποτε στοιχείο που αφορά την εκτέλεση της πράξης, εφόσον ζητηθούν.

5. ΔΗΜΟΣΙΟΤΗΤΑ

(i) Να αποδέχεται τη συμπερίληψή τους στο κατάλογο των πράξεων του Ε.Π. που δημοσιοποιεί η Ειδική Υπηρεσία Διαχείρισης του Ε.Π. (ή εναλλακτικά ο ΕΦ), στη διαδικτυακή πύλη www.espa.gr, κατά τα προβλεπόμενα στο άρθρο 115 και στο Παράρτημα XII του Καν. 1303/2013, και στο οποίο αναφέρονται: η ονομασία του δικαιούχου και της πράξης,σύνοψη της πράξης, ημερομηνία έναρξης της πράξης, καταληκτική ημερομηνία πράξης, συνολική επιλέξιμη δαπάνη, ποσοστό συγχρηματοδότησης, ταχυδρομικός κώδικας, ή άλλη κατάλληλη ένδειξη της τοποθεσίας, χώρα, ονομασία της κατηγορίας παρέμβασης τηςπράξης.

(ii) Να λαμβάνει όλα τα μέτρα πληροφόρησης που προβλέπονται στο Παράρτημα XII του Κανονισμού 1303/2013 και ειδικότερα:

α) Να αναρτά προσωρινή πινακίδα, σημαντικού μεγέθους, στο εργοτάξιο των έργων υποδομής ή κατασκευών σε ορατό σημείο από το κοινό, με συνολική δημόσια δαπάνη άνω των 500.000 ευρώ, κατά τη φάση υλοποίησής τους.

β) Να τοποθετεί μόνιμη αναμνηστική πλάκα ή πινακίδα σημαντικού μεγέθους, σε σημείο εύκολα ορατό από το κοινό, εντός τριών μηνών από την ολοκλήρωση του έργου υποδομής ή κατασκευών ή αγοράς φυσικού (ενσώματου) αντικειμένου, με συνολική δημόσια δαπάνη άνω των 500.000 ευρώ.

Οιαναμνηστικές πλάκες ή πινακίδες, οι οποίες σχεδιάζονται σύμφωνα με τα τεχνικά χαρακτηριστικά που καθορίζονται στον 821/2014, αναγράφουν την ονομασία και τον κύριο στόχο του, το έμβλημα της ένωσης μαζί με την αναφορά στην Ένωση, και το Ταμείο ή τα Ταμεία που στηρίζουν το έργο.

γ) Να λειτουργεί διαδικτυακό τόπο, στον οποίο θα αναρτά στοιχεία της πράξης, όπως σύντομη περιγραφή, ανάλογης με το επίπεδο της στήριξης, στόχοι και αποτελέσματα, επισημαίνοντας τη χρηματοδοτική στήριξηαπό την Ένωση.

δ) Να τοποθετεί αφίσες με πληροφόρηση σχετικά με την πράξη, σε πράξεις που δεν εμπίπτουν στην υποχρέωση πινακίδων ή πλακών.

ε) Να ενημερώνει τους συμμετέχοντες σε πράξεις που συγχρηματοδοτούνται από το ΕΚΤ, και κατά περίπτωση από το ΕΤΠΑ ή Ταμείο Συνοχής, σχετικά με τη συγχρηματοδότησή της από το ΕΚΤ, ή το ΕΤΠΑ ή το Τ.Σ. καιτην υλοποίησή της στο πλαίσιο επιχειρησιακού προγράμματος. Η εν λόγω ενημέρωση πραγματοποιείται σε κάθε έγγραφο ή άλλο πιστοποιητικό που χρησιμοποιούνται κατά την υλοποίηση της πράξης ή παράγονται στο πλαίσιο αυτό.

στ) Να εξασφαλίζει ότι οι συμμετέχοντες σε πράξεις που υλοποιούνται στο πλαίσιο της Πρωτοβουλίας για την Απασχόληση των Νέων (ΠΑΝ) είναι ειδικά ενημερωμένοι για την υποστήριξη της ΠΑΝ που παρέχεται από τους πόρους του ΕΚΤ και τα ειδικά κονδύλια για την ΠΑΝ. Κάθε έγγραφο που αφορά στην υλοποίηση μιαςπράξης και το οποίο απευθύνεται στο κοινό ή στους συμμετέχοντες, συμπεριλαμβανομένων των πιστοποιητικών συμμετοχής ή άλλων πιστοποιητικών, περιλαμβάνει δήλωση ότι η πράξη υποστηρίχθηκε στο πλαίσιο της ΠΑΝ.

6. ΤΗΡΗΣΗ ΣΤΟΙΧΕΙΩΝ ΚΑΙ ΔΙΚΑΙΟΛΟΓΗΤΙΚΩΝ ΑΠΟ ΔΙΚΑΙΟΥΧΟΥΣ

(i) Να τηρεί και να ενημερώνει φάκελο πράξης με όλα τα στοιχεία που αφορούν στην εκτέλεση της πράξης έως την ολοκλήρωση, την αποπληρωμή και τη λειτουργία της. Στο φάκελο της πράξης να τηρούνται όλα τα δικαιολογητικά έγγραφα σχετικά με τις δαπάνες και τους λογιστικούς ελέγχους για διάστημα δύο (2) ετών, από την 31 Δεκεμβρίου που ακολουθεί την υποβολή των λογαριασμών στους οποίους περιλαμβάνεται η τελική δαπάνη της ολοκληρωμένης πράξης. Η ΔΑ ενημερώνει τον δικαιούχο για την ημερομηνία έναρξης της περιόδου διαθεσιμότητας των εγγράφων κατά την ολοκλήρωση της πράξης. Τα ανωτέρω στοιχεία και δικαιολογητικά έγγραφα διατηρούνται είτε υπό τη μορφή πρωτοτύπων, ή επικαιροποιημένων αντιγράφων των πρωτοτύπων ήσε κοινώς αποδεκτούς φορείς δεδομένων, περιλαμβανομένων των ηλεκτρονικών εκδόσεων των πρωτότυπων εγγράφων ή εγγράφων που υπάρχουν μόνο σε ηλεκτρονική μορφή.

(ii) Να κοινοποιεί στην αρμόδια Ειδική Υπηρεσία Διαχείρισης του Ε.Π (ή εναλλακτικά στον ΕΦ) το έντυπο Ε.Ι.1_6 «Κατάσταση τήρησης φακέλου Πράξης», στο οποίο, μεταξύ άλλων, καταγράφονται τα στοιχεία ταυτότητας και η διεύθυνση των φορέων στους οποίους τηρούνται στοιχεία και έγγραφα, καθώς και η μορφή υπό την οποία θα τηρούνται, το αργότερο με την υποβολή του πρώτου Δελτίου Δήλωσης Δαπάνης.

(iii) Να τηρεί ειδικότερους όρους ή περιορισμούς που τίθενται από το ειδικό θεσμικό πλαίσιο εφαρμογής της πράξης ή που τίθενται από την Ειδική Υπηρεσία Διαχείρισης του Ε.Π (ή εναλλακτικά τον ΕΦ).

(iv) Να τηρεί τις ακόλουθες μακροχρόνιες δεσμεύσεις, προκειμένου οι πράξεις να διατηρήσουν το δικαίωμα της συνεισφοράς των Ταμείων:

α) Για πράξεις επένδυσης σε υποδομή ή παραγωγική επένδυση, εντός πέντε (5) ετών απότην τελική πληρωμή ή εντός της προθεσμίας που ορίζεται στους κανόνες περί κρατικών ενισχύσεων να μην επέλθει:

ό παύση ή μετεγκατάσταση μιας παραγωγικής δραστηριότητας εκτός της περιοχής προγράμματος

ό αλλαγή του ιδιοκτησιακού καθεστώτος ενός στοιχείου υποδομής η οποία παρέχει σε μια εταιρεία ή δημόσιοοργανισμό αδικαιολόγητο πλεονέκτημα

ό ουσιαστική μεταβολή που επηρεάζει τη φύση, τους στόχους ή την εφαρμογή των όρων που θα μπορούσαν να υπονομεύσουν τους αρχικούς στόχους.

β) Άλλες μακροχρόνιες δεσμεύσεις που ορίζονται από την Ειδική Υπηρεσία Διαχείρισης του Ε.Π (ή εναλλακτικά τον ΕΦ) ή καθορίζονται από το θεσμικό πλαίσιοπου διέπει την πράξη.

Η τήρησητων μακροχρονίων υποχρεώσεων επιβεβαιώνονται, μετά την ολοκλήρωση τηςπράξης, με διοικητικές ή και επιτόπιες επαληθεύσεις από την Δ.Α. ή τον ΕΦ. (Στην περίπτωση που η υποχρέωση τήρησης των μακροχρονίων δεσμεύσεων μεταβιβάζεται σε άλλο φορέα, αυτός προσδιορίζεται και η υποχρέωση αφορά αυτόν τον φορέα)

Η τήρηση των μακροχρονίων υποχρεώσεων επιβεβαιώνονται, μετά την ολοκλήρωση της πράξης, με διοικητικές ή και επιτόπιες επαληθεύσεις από την Δ.Α. ή τον ΕΦ. (Στην περίπτωση που η υποχρέωση τήρησης τωνμακροχρονίων δεσμεύσεων μεταβιβάζεται σε άλλο φορέα, αυτός προσδιορίζεται και η υποχρέωση αφορά αυτόν τον φορέα)

7. Ειδικοί Όροι

Η χρηματοδότηση της πράξης θα εξαρτάται άμεσα από την πρόοδο συλλογής και συμπλήρωσης των Δεδομένων Συμμετεχόντων (microdata) από τον Δικαιούχο σύμφωνα με όσα προβλέπονται στο Κανονιστικό Πλαίσιο.

Αφορά τις πράξεις που συγχρηματοδοτούνται από το ΕΚΤ/ΠΑΝ, για τις οποίες απαιτείται η συλλογή δεδομένων μεμονωμένων συμμετεχόντων (microdata) μέσω των ερωτηματολογίων που καλούνται να συμπληρώσουν οι ωφελούμενοι συμμετέχοντες κατά την είσοδο και έξοδό τους από τις πράξεις. Οι δικαιούχοι των εν λόγω πράξεων, πέραν των γενικών υποχρεώσεων δικαιούχων (Παράρτημα Ι), αναλαμβάνουν επιπλέον την τήρηση των παρακάτω υποχρεώσεων:

Α. Χρονικό Πλαίσιο υποβολής στοιχείων στο ΟΠΣ

Να υποβάλλουν στο ΟΠΣ το σύνολο όλων των συλλεχθέντων απογραφικών δελτίων εισόδου και εξόδου για κάθε ωφελούμενο συμμετέχοντα, το αργότερο εντός 30 ημερών από την είσοδο και έξοδο των συμμετεχόντων, αντίστοιχα.
Να υποβάλλουν το Δελτίο Δήλωσης Επίτευξης Δεικτών Πράξης κάθε Έτος.

Β. Συλλογή δεδομένων
1. Να εξασφαλίσουν ότι οι συμμετέχοντες ενημερώνονται σχετικά με τις υποχρεώσεις και τα δικαιώματα τους σε ό,τι αφορά την απαιτούμενη ορθή συμπλήρωση του συνημμένου ερωτηματολογίου/ απογραφικού δελτίου και ειδικότερα για τους σκοπούς για τους οποίους τηρούνται τα δεδομένα τους, καθώς και για τις ρυθμίσεις που εφαρμόζονται για την προστασία τους από τους φορείς που τα επεξεργάζονται.
2. Να εξασφαλίσουν ότι κάθε συμμετέχων/ωφελούμενος συμπλήρωσε απογραφικό δελτίο εισόδου, κατά την έναρξη συμμετοχής του (είσοδο) στην πράξη και απογραφικό δελτίο εξόδου κατά την έξοδό του από αυτήν.
Ως είσοδος στην πράξη (έναρξη συμμετοχής) ορίζεται η ημερομηνία έναρξη της Σύμβασης του ωφελούμενου.
Ως έξοδος από την πράξη (ολοκλήρωση/λήξη της συμμετοχής) ορίζεται η ημερομηνία λήξης Σύμβασης του ωφελούμενου.
Η συλλογή των απογραφικών δελτίων εισόδου και εξόδου (συμπληρωμένα ερωτηματολόγια) σε έντυπη και ηλεκτρονική μορφή διενεργείται από τον Δικαιούχο.
3. Να προβαίνουν σε όλες τις απαιτούμενες διορθωτικές ενέργειες προκειμένου να εξασφαλίζεται η συμπλήρωση των απογραφικών δελτίων με πληρότητα και εγκυρότητα σε όλα τα πεδία, πριν την υποβολή τους στο ΟΠΣ.
4. Να διατηρούν τα δεδομένα/απογραφικά δελτία στο σύστημα πρώτης καταχώρησης που είναι εγκατεστημένο στον Δικαιούχο ή τα πρωτότυπα συμπληρωμένα έντυπα για διάστημα δύο (2) ετών από την 31 Δεκεμβρίου που ακολουθεί την υποβολή των λογαριασμών στους οποίους περιλαμβάνεται η τελική δαπάνη της ολοκληρωμένης πράξης.

ΙΑΤΡΙΚΕΣ & ΒΙΟΛΟΓΙΚΕΣ ΕΠΙΣΤΗΜΕΣ

ιθμός αίτης	Τίτλος
1629	Ο ρόλος της ογκοκατασταλτικής πρωτεΐνης CYLD στην διαφοροποίηση επιθηλιακών κυττάρων μαστού σε λιποκύτταρα.
2056	ΧΡΗΣΗ ΤΩΝ ΠΤΗΤΙΚΩΝ ΟΡΓΑΝΙΚΩΝ ΕΝΩΣΕΩΝ (VOCS) ΣΤΟΝ ΕΚΠΝΕΟΜΕΝΟ ΑΕΡΑ ΓΙΑ ΤΗΝ ΑΝΑΓΝΩΡΙΣΗ ΑΣΘΕΝΩΝ ΜΕ ΧΑΠ ΩΣ ΥΨΗΛΟΥ ΚΙΝΔΥΝΟΥ ΓΙΑ ΠΑΡΟΞΥΝΣΕΙΣ(VOCS FOR THE IDENTIFICATION OF HIGH RISK COPD PATIENTS)
1110	Εκτίμηση της χωρικής συμπεριφοράς της αγριόγατας (Felis silvestris silvestris) με χρήση τηλεμετρίας σε προστατευόμενες περιοχές της Βόρειας Ελλάδας
1980	Εκπαίδευση στην Αναζωογόνηση σε μαθητές γυμνασίου: η επίδραση της εκπαίδευσης από ισάξιους (peer - education) στην εκμάθηση της τεχνικής και στο αίσθημα αυτο-αποτελεσματικότητας (self-efficacy)
95	Μελέτη της λειτουργικής σύνδεσης της γενωμικής αστάθειας με τον μεταβολισμό του RNA κατά την ανάπτυξη των θηλαστικών.
105	ΟΛΟΚΛΗΡΩΜΕΝΟ ΠΡΟΤΥΠΟ ΜΕΛΕΤΗΣ ΓΙΑ ΤΗΝ ΑΛΛΟΧΘΟΝΗ ΧΛΩΡΙΔΑ
1008	"Διερεύνηση της ευεργετικής επίδρασης της μικρονευροτροφίνης BNN-20 σε νευρώνες που προέρχονται από βλαστικά κύτταρα ασθενών με νόσο Πάρκινσον και στην επιτυχία μεταμοσχεύσεων βλαστοκυττάρων σε ζωικό μοντέλο της νόσου."
979	Ανάπτυξη Μεθόδου για την Ανίχνευση Βακτηριακών και Ιογενών Λοιμώξεων του Αναπνευστικού με βάση τις Τεχνολογίες Αλληλούχισης 3ης Γενεάς
275	Διερεύνηση της χρήσης ελεύθερων νουκλεϊνικών οξέων (cell-free DNA & miRNAs) στα καλλιεργητικά υλικά εμβρύων ως μη επεμβατικό εργαλείο προεμφυτευτικής διάγνωσης
659	Ρόλος της πρωτεΐνης PML στη βιολογία φυσιολογικών και καρκινικών βλαστοκυττάρων.
99	Επιβεβαίωση-ενίσχυση της προγνωστικής σημασίας του βήτα υποδοχέα οιστρογόνων στον πρώιμο καρκίνο του μαστού.
355	Μελέτη της συμμετοχής της πλειοτροπίνης στην αναδιαμόρφωση των οστών με στόχο την ανάδειξη νέων θεραπευτικών στόχων και/ή προσεγγίσεων
2073	Ο ρόλος των βιομορίων στο σάλιο και στο υγρό της ουλοδοντικής σχισμής ως δεικτών εκτίμησης της σκελετικής ωρίμανσης στα παιδιά
65	Αξιολόγηση της πιθανής ευεργετικής επίδρασης της Μεσογειακής Διατροφής ως πρότυπου αειφόρου διατροφής στην ψυχική υγεία και την ευζωία του φοιτητικού πληθυσμού των ελληνικών πανεπιστημιακών ιδρυμάτων
1249	Διερεύνηση του ρόλου της αυτοφαγίας ως ρυθμιστή των υπέρμετρων αποκρίσεων σε ασθενείς με σοβαρό άσθμα.
104	Αποκωδικοποίηση παραμέτρων της οπτικής προσοχής και της συμπεριφοράς από νευρωνικά σήματα
139	Μελέτη του ρόλου των εισοσωμικών πρωτεϊνών στην κατάσταση αδράνειας των μυκήτων
2047	Ψηφιακός Εθισμός: Αναγνώριση και Καθοδήγηση Θεραπευτικών Παρεμβάσεων Μέσω Ανίχνευσης του Φυσικού και Κοινωνικού Περιβάλλοντος
1380	ΕΠΕΚΤΑΣΗ ΤΗΣ ΜΕΤΑ-ΑΝΑΛΥΣΗΣ ΔΙΚΤΥΩΝ ΓΙΑ ΤΗ ΣΥΓΚΡΙΣΗ ΔΙΑΓΝΩΣΤΙΚΩΝ ΤΕΣΤ
290	Στεροειδείς ορμόνες του φύλου και καρκίνος: μελέτη μοριακής επιδημιολογίας
33	Επιμήκης Άξονας Ιπποκάμπου Επίμυος: Ρύθμιση Συναπτικής Διαβίβασης - Πλαστικότητας & Ρυθμογένεση στη Φυσιολογία και τον Αυτισμό.

753	Ανάπτυξη κονιών συγκόλλησης ζιρκονίας με νανοσωματίδια σταθεροποιημένης ζιρκονίας: φυσικοχημικές ιδιότητες και αντοχή δεσμού σε συνθήκες γήρανσης.
1147	Μελέτη των Δυναμικών Μηχανισμών Δημιουργίας,Διατήρησης και Ανάκλησης της Ανοσολογικής Μνήμης Μετά από Εμβολιασμό: Η επίτευξη της μακρόβιας προστασίας
96	Κατευθυνόμενος από τη δομή σχεδιασμός ρυθμιστών της γλυκογονόλυσης με στόχο την ανάπτυξη νέων θεραπευτικών μέσων
1137	Σιδηρομαγνητικά νανοσωματιδία διπλής στόχευσης κατά των υποδοχέων μπομπεσίνης (BN) και του ειδικό προστατικό μεβρανικό αντιγόνο (PSMA), επισημασμένα με Γάλλιο-68 ως PET/MRI απεικονιστικοί παράγοντες καρκίνου του προστάτη
999	Μελέτη της συσσώρευσης αντικαρκινικών ενώσεων σε ιστούς μεσοσπονδύλιου δίσκου και επίδρασή τους στην κυτταρική γήρανση
673	Πηγές μεταβλητότητας στη λειτουργική ποικιλότητα των βενθικών κοινοτήτων και η λειτουργική πλεονασματικότητα (functional redundancy) των ειδών ως δείκτης της ακεραιότητας του θαλάσσιου πυθμένα
563	Κυτταρικοί διαμεσολαβητές που τροποποιούν την επίκτητη ανοσιακή απόκριση κατά την HCV λοίμωξη
1037	Μεταβολομική ανάλυση περιφερικού αίματος ως δυνητικός βιοδείκτης σε ασθενείς με Πολλαπλή Σκλήρυνση με κλινική και/ή υποκλινική ενεργότητα.
1083	Μηχανισμοί μετάδοσης και αποικοδόμησης τοξικών μορφών της α-συνουκλεΐνης
742	Ο ρόλος των long non-coding RNAs στον καθορισμό της ταυτότητας των Νευρικών Βλαστικών Κυττάρων κατά την ανάπτυξη
636	Η επίδραση παρέμβασης σε παραμέτρους του τρόπου ζωής στο καρδιομεταβολικό προφίλ ασθενών με αποφρακτική άπνοια ύπνου.
1838	Η επίδραση της χορήγησης κινολονών στην αύξηση της διαμέτρου της αορτής, στην αορτική σκληρία και στα ανακλώμενα κύματα [The effect of FluoRoquinolones on Aortic Growth, aortic stIffness and wave refLEctionS - FRAGILES trial]
1396	Γενετική προδιάθεση και ρύθμιση σωματικού βάρους. Αξιολόγηση των γονιδίων στόχων μετά από διατροφικές παρεμβάσεις σε πληθυσμό ενήλικων υπέρβαρων/παχύσαρκων εθελοντών.
1039	Ο ΡΟΛΟΣ ΤΗΣ ΟΓΚΟΚΑΤΑΣΤΑΛΤΙΚΗΣ ΠΡΩΤΕΪΝΗΣ CYLD ΣΤΗΝ ΑΝΑΠΤΥΞΗ ΝΕΟΠΛΑΣΙΩΝ ΝΕΦΡΟΥ
987	Νεφρικές σωληναριακές αλλοιώσεις στην υπέρταση
1164	ΔΙΕΡΕΥΝΗΣΗ ΤΗΣ ΑΛΛΗΛΕΠΙΔΡΑΣΗΣ ΤΩΝ ΑΝΑΠΤΥΞΙΑΚΩΝ ΜΟΝΟΠΑΤΙΩΝ mTOR ΚΑΙ HIPPO ΣΤΗΝ ΠΟΡΕΙΑ ΩΡΙΜΑΝΣΗΣ ΤΩΝ ΩΟΘΥΛΑΚΙΩΝ ΚΑΙ Η ΑΞΙΟΠΟΙΗΣΗ ΤΟΥΣ ΣΕ ΤΕΧΝΙΚΕΣ ΥΠΟΒΟΗΘΟΥΜΕΝΗΣ ΑΝΑΠΑΡΑΓΩΓΗΣ
1768	Ανάπτυξη μικροβελόνων με την χρήση τρισδιάστατης εκτύπωσης για εξατομικευμένη διαδερμική χορήγησης ινσουλίνης
551	Διερεύνηση της Μοριακής Δράσης του miR-16-5p στη Βιογένεση των Ριβοσωμάτων και Φαρμακολογική Αξιοποίησή του στη Θεραπεία του Καρκίνου
1269	Δημιουργία διακριτών τύπων τοιχωματικών αγγειακών κυττάρων (Mural Cells-MCs) από τη διαφοροποίηση ανθρώπινων πολυδύναμων βλαστικών κυττάρων και εφαρμογή τους στην κατασκευή μιας αγγειωμένης ιστικής κατασκευής
1673	Η επίδραση της Κεταμίνης στη γλουταματεργική νευροδιαβίβαση και νευρωνική πλαστικότητα. Διερεύνηση πιθανής ανασχετικής δράσης της Κανναβιδιόλης.
27	Μελέτη της νέας αλληλεπίδρασης του HIF-2α με τη Ρεπτίνη και ο ρόλος της στην προσαρμογή των καρκινικών κυττάρων στην υποξία.

1945	ΜΕΛΕΤΗ ΤΗΣ ΕΠΙΔΡΑΣΗΣ ΤΗΣ ΕΝΔΑΓΓΕΙΑΚΗΣ ΑΠΟΚΑΤΑΣΤΑΣΗΣ ΑΝΕΥΡΥΣΜΑΤΩΝ ΚΟΙΛΙΑΚΗΣ ΑΟΡΤΗΣ ΣΤΗΝ ΠΡΟΚΛΗΣΗ ΟΞΕΙΑΣ ΝΕΦΡΙΚΗΣ ΒΛΑΒΗΣ ΣΕ ΑΣΘΕΝΕΙΣ ΠΟΥ ΠΑΣΧΟΥΝ ΑΠΟ ΑΝΕΥΡΥΣΜΑ ΚΑΙ ΥΠΟΒΑΛΛΟΝΤΑΙ ΣΕ ΕΠΕΜΒΑΣΗ ΕΝΔΑΓΓΕΙΑΚΗΣ ΑΝΤΙΜΕΤΩΠΙΣΗΣ
1178	ΕΜΒΟΛΙΟ-on-chip ΕΝΑΝΤΙΑ ΣΤΗ ΣΗΨΗ
1590	Ο μεταγραφικός παράγοντας ERF ρυθμίζει τη διαφοροποίηση των CD4+ T βοηθητικών κυττάρων.
97	Πιάνοντας με το χέρι και το νου: Είναι η νευρική δραστηριότητα που επάγεται από την παρατήρηση μιας κίνησης επαρκής για τον έλεγχο κινητικής δι-επαφής ικανής να εκτελεί επιδέξιες λαβές σύλληψης αντικειμένων;
119	Μελέτη της βελτιστοποίησης εξελιγμένων τεχνικών ακτινοθεραπείας όγκων εγκεφάλου με χρήση νανοσωματιδίων χρυσού.
1253	Διερεύνηση της οξείας και χρόνιας φυσιολογικής και συμπεριφορικής απόκρισης μικρών θηλαστικών σε στρεσσογόνα ερεθίσματα, με χρήση μη-επεμβατικών μεθόδων σε συνθήκες δειγματοληψίας πεδίου
1578	Μελέτη βιοδραστικών συστατικών από φυτά της ελληνικής βιοποικιλότητας ως διατροφική πηγή για την πρόληψη και αντιμετώπιση του παιδικού καρκίνου• έμφαση στο saffron και τους δευτερογενείς μεταβολίτες του (κροκίνες, κροκετίνη, διμεθυλοκροκετίνη).
1618	ΣΗΜΑΣΙΑ ΤΩΝ ΕΝΔΟΤΟΙΧΩΜΑΤΙΚΩΝ ΜΙΚΡΟΑΙΜΟΡΡΑΓΙΩΝ ΚΑΙ ΤΗΣ ΕΡΥΘΡΟΚΥΤΤΑΡΙΚΗΣ ΛΥΣΗΣ ΣΤΗΝ ΑΓΓΕΙΑΚΗ ΕΠΑΣΒΕΣΤΩΣΗ ΚΑΙ ΑΘΗΡΟΣΚΛΗΡΩΣΗ: ΡΟΛΟΣ ΤΟΥ ΕΡΥΘΡΟΚΥΤΤΑΡΙΚΟΥ ΜΟΝΟΞΕΙΔΙΟΥ ΤΟΥ ΑΖΩΤΟΥ
1091	Μελέτη ανθρώπινων φυσικών μονοκλωνικών αντισωμάτων με ικανότητα κυτταροδιείσδυσης ως μοντέλο στοχευμένης και συνδυαστικής αντικαρκινικής θεραπείας.
643	Πρωτεασωμική Ενεργοποίηση: Κατανόηση της συστημικής ρύθμισης της πρωτεόστασης κατά τη γήρανση και ταυτοποίηση αντιγηραντικών δομικών πρωτεασωμικών ενεργοποιητών από τη Μεσόγειο Θάλασσα (ακρωνύμιο: Proteostasis)
1725	Διερεύνηση της προφυλακτικής δράσης της ταμσουλοσίνης στην μετεγχειρητική εμφάνιση επίσχεσης ούρων μετά από πλαστική αποκατάσταση βουβωνοκήλης υπό ραχιαία αναισθησία και διερεύνηση περιεγχειρητικών παραγόντων κινδύνου. Προοπτική τυχαιοποιημένη διπλή τυφλή μελέτη
719	Διερεύνηση της επιδημίας HIV και ηπατίτιδας C στον πληθυσμό των χρηστών ενδοφλέβιων ναρκωτικών στην Ελλάδα και ανάδειξη των αποτελεσματικότερων παρεμβάσεων με εφαρμογή μαθηματικών μοντέλων και μεθόδων μοριακής επιδημιολογίας
595	Ο ρόλος της SIRT-1 στην οστεοαρθρίτιδα: λειτουργική συσχέτιση μεταβολισμού λιπιδίων - αυτοφαγίας και διερεύνηση των εμπλεκόμενων μηχανισμών επιγενετικής ρύθμισης
1419	ΜΕΛΕΤΗ ΔΙΑΦΟΡΙΚΗΣ ΕΜΠΛΟΚΗΣ ΙΣΟΜΟΡΦΩΝ ΤΗΣ ΝΕΥΡΩΝΙΚΗΣ ΠΡΩΤΕΙΝΗΣ TAU ΣΕ ΚΙΝΗΤΙΚΕΣ ΚΑΙ ΓΝΩΣΙΑΚΕΣ ΝΟΣΗΡΟΤΗΤΕΣ ΤΑΥΠΑΘΕΙΩΝ IN VIVO.
935	Μεταμόσχευση βλαστοκυττάρων σε ζωϊκό μοντέλο πολλαπλής σκλήρυνσης με στόχο την αποτελεσματική κυτταρική θεραπεία
1365	Μεταβολικό αποτύπωμα πρωτογάλατος μητέρων με υποθυρεοειδισμό στο στάδιο της γαλουχίας
1364	Χαρτογράφηση του πρωτεύοντα κινητικού φλοιού του ανθρώπου με διακρανιακό μαγνητικό ερεθισμό και νευροπλοήγηση (navigated TMS, n TMS): βελτιστοποίηση της μεθόδου με εφαρμογή προηγμένων νευροφυσιολογικών μεθόδων και υπολογιστικών τεχνικών
334	Η μεθυλίωση του DNA ως ελάχιστα επεμβατικός βιοδείκτης: ανάπτυξη και επικύρωση ταξινομητών με προγνωστική ή/και προβλεπτική αξία στη φαρμακοθεραπεία του καρκίνου του μαστού.
1016	Επιδράσεις της ατμοσφαιρικής ρύπανσης σε ασθενείς με ιδιοπαθή πνευμονική ίνωση

945	Σχεδιασμός, Χαρακτηρισμός και Μελέτη Φαρμακολογικά Τροποποιημένου Ενδοφακού-Ενδοφθάλμιου Εμφυτεύματος με Σκοπό την Χειρουργική Αντιμετώπιση του Καταρράκτη και της Μετεγχειρητικής Φλεγμονής - in vitro & in vivo μελέτη
713	Ανάπτυξη μεθοδολογίας για την αλληλούχιση ολόκληρου του γονιδιώματος στελεχών ιού του Δυτικού Νείλου με την εφαρμογή της τεχνολογίας επόμενης γενιάς
1477	Ανάπτυξη βιοδεικτών για τη διάγνωση της νόσου Πάρκινσον.
164	Στόχευση της μεταβολικής οδού της κυνουρενίνης για απεικόνιση και χαρακτηρισμό καρκινικών όγκων μέσω τομογραφίας εκπομπής φωτονίου (SPECT) και ποζιτρονίου (PET)
1145	Ανάπτυξη αυτοματοποιημένης δοκιμασίας ποιοτικού ελέγχου βραχυθεραπείας, βάσει τμηματοποιημένων θαλάμων ιονισμού
1171	Η φυλογεωγραφία στη γενομική εποχή και ο ρόλος των φραγμάτων στην ειδογένεση.
1669	Μελέτη έκφρασης μικρών μη-κωδικών μορίων RNA στο πολλαπλό μυέλωμα και συσχέτισή τους με την οστική νόσο.
**855**	**NeuroSuitUp: Νευροαποκατάσταση μέσω συνεργικών διεπαφών ανθρώπου-μηχανής, προάγοντας την αδρανή νευροπλαστικότητα στην κάκωση νωτιαίου μυελού.**
2080	ΣΥΓΚΡΙΤΙΚΗ ΜΕΛΕΤΗ ΤΗΣ ΜΗ ΕΝΖΥΜΑΤΙΚΗΣ ΓΛΥΚΟΖΥΛΙΩΣΗΣ ΣΕ ΑΟΡΤΙΚΕΣ ΒΑΛΒΙΔΕΣ ΑΣΘΕΝΩΝ ΜΕ ΑΟΡΤΙΚΗ ΣΤΕΝΩΣΗ ΚΑΙ ΖΩΙΚΟΥ ΠΡΟΤΥΠΟΥ ΚΟΝΙΚΛΩΝ NEW ZEALAD
445	ΜΕΛΕΤΗ «ΦΕΙΔΙΠΠΙΔΗΣ»: ΚΑΡΔΙΑΓΓΕΙΑΚΕΣ ΕΠΙΔΡΑΣΕΙΣ ΤΟΥ ΜΑΡΑΘΩΝΙΟΥ
1115	Αξιολόγηση νεότερων δεικτών ενδοθηλιακής δυσλειτουργίας και θρομβωτικού μικροπεριβάλλοντος σε ασθενείς με ρευματοειδή αρθρίτιδα: συσχέτιση με δείκτες υποκλινικής φλεγμονής και καρδιαγγειακής βλάβης
415	Η επίδραση των συνθηκών φωτισμού στην αποτελεσματικότητα της χειρουργικής διόρθωσης της πρεσβυωπίας
1221	Μεθοδολογικό πλαίσιο για την ανάπτυξη και επικαιροποίηση εξατομικευμένων και τεκμηριωμένων κατευθυντήριων οδηγιών. Εφαρμογή στον σακχαρώδη διαβήτη τύπου 2.
626	Μεταβολισμός του Παράγοντα Ενεργοποίησης Αιμοπεταλίων (PAF) σε φαινομενικά υγιείς εθελοντές μετά από διατροφική παρέμβαση με τρόφιμο εμπλουτισμένο με αναστολέα του PAF φυσικής προέλευσης
1084	Αιτιατά Μοντέλα Πρόβλεψης στις Επεμβατικές Μεθόδους Αντιμετώπισης των Χρόνιων Ολικών Αποφράξεων των Στεφανιαίων Αγγείων
996	Ανάπτυξη μοντέλου πρόβλεψης επιπλοκών στην κύηση χρησιμοποιώντας δεδομένα των εγκύων από την ιατρική τους παρακολούθηση και από την καθημερινότητα τους.
199	Φαρμακοδυναμικές και ανοσοτροποποιητικές δράσεις αντιμυκητιακών φαρμάκων έναντι βιοϋμενίων κλινικά σημαντικών υφομυκήτων
928	Πρόβλεψη εμφάνισης επιβλαβών εξάρσεων μικροφυκών με χρήση μεθόδων μηχανικής μάθησης
1324	Η επίδραση της ενδοπεριτοναϊκής έγχυσης δύο νέων χημειοθεραπευτικών παραγόντων, της ρεγοραφενίμπης και της τριφλουριδίνης/τιπιρασίλης, στην περιτοναϊκή διασπορά του καρκίνου του παχέος εντέρου μετά κυτταρομείωση σε επίμυες.
1643	Λειτουργικός ρόλος της κινάσης Haspin στα εμβρυονικά βλαστικά και γαμετικά κύτταρα.

1798	Επίδραση της θερμοκρασίας στην εμφάνιση παραμορφώσεων στα εμβρυικά στάδια του προστατευόμενου θαλάσσιου γαστερόποδου Charonia seguenzae (Aradas & Benoit, 1870).
714	Μελέτη της διατροφικής πρόσληψης σακχάρων (μονο - και δι - σακχαριτών) σε πληθυσμούς με παράγοντες καρδιαγγειακού κινδύνου ή/και χρόνια φλεγμονώδη νοσήματα: ποσοτική ανάλυση και συσχέτιση με την υποκλινική αγγειακή βλάβη σε επίπεδο μικρο - και μακρο - κυκλοφορίας
1420	Η συνδυαστική επίδραση συνιστωσών του τρόπου ζωής στην άνοια και στην έκπτωση των γνωσιακών λειτουργιών: 3ετής επανέλεγχος της μελέτης HELIAD
71	Μελέτη της επίδρασης αυξητικών παραγόντων επί της κινητικότητας και ζωτικότητας ανθρώπινων σπερματοζωαρίων
1481	Ανατομική μελέτη παραλλαγών της πρόσφυσης του έσω επιγονατιδομηριαίου συνδέσμου. Απεικονιστική και εμβιομηχανική αποτίμηση των τεσσάρων πιο διαδεδομένων τεχνικών ανακατασκευής του, σε περιπτώσεις οξείας ρήξης του.
904	Μελέτη των δυναμικών αλληλεπιδράσεων μεταξύ του ρυθμού έκλυσης των γλυκοκορτικοειδών και του νευροτροφικού παράγοντα BDNF υπό φυσιολογικές και παθολογικές συνθήκες: in vitro μελέτη σε νευρώνες και γλοιακά κύτταρα.
2031	Έκφραση του γονιδίου hTERT σε ασθενείς με καρκίνο πνεύμονα και συνυπάρχουσα ιδιοπαθή πνευμονική ίνωση.
1112	Χωρικά πρότυπα μεταβολών της λειτουργικής και φυλογενετικής ποικιλότητας στο χρόνο και οι παράγοντες που τα επηρεάζουν
1312	«Η επίδραση της εφαρμογής ενός συνδυαστικού προγράμματος κινητικού ελέγχου και εργονομικής βελτίωσης της οικιακής ασφάλειας στη μείωση των πτώσεων σε ευπαθή περιπατητικά ηλικιωμένα άτομα»
963	Ανάπτυξη in vitro μοντέλου εντερικής ζύμωσης για την μελέτη των Διαταραχών Φάσματος του Αυτισμού
1320	ΟΛΙΣΤΙΚΗ ΔΙΕΠΙΣΤΗΜΟΝΙΚΗ ΠΡΟΣΕΓΓΙΣΗ ΣΤΗΝ ΑΝΤΙΜΕΤΩΠΙΣΗ ΑΣΘΕΝΩΝ ΜΕ ΚΑΤΑΓΜΑΤΑ ΕΥΘΡΑΥΣΤΟΤΗΤΑΣ
717	ΜΕΛΕΤΗ ΤΗΣ ΜΙΤΟΧΟΝΔΡΙΑΚΗΣ ΠΡΩΤΕΙΝΗΣ SLC25A46 ΣΤΗΝ ΠΑΘΟΦΥΣΙΟΛΟΓΙΑ ΝΕΥΡΟΕΚΦΥΛΙΣΤΙΚΩΝ ΑΣΘΕΝΕΙΩΝ ΣΕ ΓΕΝΕΤΙΚΟ ΜΟΝΤΕΛΟ ΠΟΝΤΙΚΟΥ
533	Ανάπτυξη αναλυτικών μεθόδων για τον προσδιορισμό κυκλικών ολιγομερών από υλικά συσκευασίας σε τρόφιμα, εκτίμηση της έκθεσης των καταναλωτών και μελέτη τοξικότητας σε κυτταρικές καλλιέργειες
801	ΜΙΚΡΟΠΕΡΙΒΑΛΛΟΝ ΣΤΟΝ ΚΑΡΚΙΝΟ ΤΟΥ ΠΝΕΥΜΟΝΑ ΚΑΙ ΕΠΙΔΡΑΣΗ ΣΤΗΝ ΚΑΤΑΣΤΟΛΗ ΤΗΣ ΑΝΟΣΟΛΟΓΙΚΗΣ ΑΝΤΙΔΡΑΣΗΣ
329	Εκτίμηση ροών άνθρακα σε δασικές συστάδες Pinus brutia Ten. διαφορετικής μεταπυρικής ηλικίας.
1568	ΜΕΛΕΤΗ ΤΗΣ ΚΟΛΙΣΤΙΝΗΣ ΣΤΑ ΠΛΑΙΣΙΑ ΤΗΣ ΕΝΙΑΙΑΣ ΥΓΕΙΑΣ
1241	Μελέτη της μηχανορυθμιζόμενης ενδοκυττάρωσης των ιντεγκρινών
1813	Η επίδραση 12 εβδομάδων ελεγχόμενης και αυτοεπιλεγόμενης άσκησης σε συνδυασμό με τη θεραπεία CPAP σε ασθενείς με Σύνδρομο Αποφρακτικής Άπνοιας στον Ύπνο στα υποπεδία των εκτελεστικών λειτουργιών του εγκεφάλου, στα επίπεδα της 25(OH)D και των δεικτών οξειδωτικού stress
235	Η κρίσιμη ισορροπία της επιληπτικής κρίσης
874	Η επίδραση της περιεγχειρητικής ενδοφλέβιας χορήγησης δεξμεδετομιδίνης ή λιδοκαΐνης στον μετεγχειρητικό πόνο, την κατανάλωση αναλγητικών, τη λειτουργία του εντέρου και την ανάνηψη μετά από γυναικολογική επέμβαση κοιλίας: τυχαιοποιημένη διπλή τυφλή μελέτη

646	«Επίδραση της in vitro άσκησης στη φαιοποίηση του λευκού λιπώδους ιστού»
1429	Δημιουργία και πιλοτική εφαρμογή εκπαιδευτικού παρεμβατικού προγράμματος Εγγραμματοσύνης της Υγείας και Εγγραμματοσύνης της Διατροφής σε ασθενείς με Υπέρταση: τυχαιοποιημένη ελεγχόμενη δοκιμή
1328	Χρήση της τεχνολογίας της τριδιάστατης εκτύπωσης για την καθοδήγηση της κατάλυσης σε ασθενείς που υποβάλλονται σε κατάλυση της κολπικής μαρμαρυγής<br>[3D-printing for ablation Guidance in patients undergoing Atrial fibriLlation Ablation - 3D GALA trial]
816	Αναδρομική μελέτη με δεδομένα της καθημερινής κλινικής πρακτικής (real world data), για την εξειδίκευση των στόχων της βέλτιστης γλυκαιμικής ρύθμισης ασθενών σε μονάδες εντατικής θεραπείας, με στόχο τη μείωση συννοσηρότητας και θνητότητας.
727	Μικροδοσιμετρία με τον κώδικα Monte Carlo GEANT4-DNA για υπολογισμούς RBE μη-συμβατικών ακτινοβολιών στην ακτινοθεραπεία
1425	Χαρτογράφηση του ρόλου των υπομονάδων του ανθρώπινου ριβονουκλεοπρωτεϊνικού συμπλόκου RNase P στη ρύθμιση της μεταγραφής και της μετάφρασης με τη χρήση εργαλείων γονιδιωματικής επεξεργασίας
218	Αξιολόγηση και καθορισμός των χωρικών αβεβαιοτήτων που εισάγει η χρήση της Απεικόνισης Μαγνητικού Συντονισμού στον καθορισμό των όγκων-στόχων και της επίδρασης τους στο σχεδιασμό στερεοτακτικής ακτινοχειρουργικής-ακτινοθεραπείας σε ασθενείς με πολλαπλές εγκεφαλικές μεταστάσεις
269	Επαγωγή της βιοσύνθεσης αντιβιοτικών μέσω συγκαλλιέργειας μικροοργανισμών και διερεύνηση γονιδιακών συστοιχιών του δευτερογενούς μεταβολισμού
584	Ποιοτική διερεύνηση του ηθικού αδιεξόδου (moral distress) των ιατρών και του ηθικού αδιεξόδου (moral distress) και ηθικού «σχίσματος» ("moral schism") των γονέων σε μονάδες εντατικής θεραπείας νεογνών.
1772	Τίτλος έρευνας: ' Ο ρόλος της Μικρο-Αλβουμινουρίας (MAB) ως βιοδείκτη στην πρόβλεψη των παροξύνσεων και των καρδιαγγειακών συμβαμάτων σε ασθενείς με Χρόνια Αποφρακτική Πνευμονοπάθεια. Μία προοπτική μελέτη' .
1808	Ακούσια Νοσηλεία Ψυχιατρικών Ασθενών στην Αθήνα: κοινωνικοί, κλινικοί και συστημικοί προγνωστικοί παράγοντες
298	ΚΑΤΕΥΘΥΝΟΜΕΝΗ ΑΠΟ ΤΗ ΔΟΜΗ ΑΝΑΚΑΛΥΨΗ ΡΥΘΜΙΣΤΙΚΩΝ ΜΟΡΙΩΝ ΓΙΑ ΤΗ ΔΡΑΣΗ ΤΗΣ ΑΜΙΝΟΠΕΠΤΙΔΑΣΗΣ IRAP
1484	Εξερευνώντας την αναπτυξιακή θεωρία για το σύνδρομο πολυκυστικών ωοθηκών: ο ρόλος των αλλαγών στην πλακουντιακή έκφραση γονιδίων και τη μεθυλίωση του εμβρυικού DNA.
1655	Ανάπτυξη προβλεπτικού αλγορίθμου ανταποκρίσεως στη θεραπεία σε ασθενείς με ψωρίαση και ψωριασική αρθρίτιδα βασισμένου σε κλινικούς, ορολογικούς ανοσολογικούς, και γενετικούς βιοδείκτες
1604	Συσχέτιση του συστήματος λιπιδίων και λιποπρωτεϊνών με την εμφάνιση και την εξέλιξη του πολλαπλού μυελώματος: ο ρόλος της HDL
902	SRPK1 κινάση: Ένας νέος ρόλος στην απόπτωση καρκινικών κυττάρων
1743	Διερεύνηση του ρόλου της πολυ(Α)-εξειδικευμένης ριβονουκλεάσης στην κιρκάδια γονιδιακή έκφραση
814	Μελέτη διερεύνησης για την συμβολή των φλεγμονωδών βακτηριακών συστατικών στη νόσο Αλζχάϊμερ

323	Συναπτικοί και ιοντικοί μηχανισμοί στον προμετωπιαίο φλοιό που υποστηρίζουν την εκμάθηση διαδικασιών μνήμης εργασίας
520	Πολυπαραγοντική αξιολόγηση καρκίνου του μαστού μέσω εξελιγμένων τεχνικών απεικόνισης και ανάπτυξη λογισμικού διαφορικής διάγνωσης με χρήση συστημάτων τεχνητής νοημοσύνης
318	Μηχανισμοί Ανάπτυξης του Γυναικείου Άνθους: Πώς ο μεταγραφικός παράγοντας SPATULA επηρεάζει τη συνεργιστική δράση της ακετυλίωσης των ιστονών και της σηματοδότησης CLAVATA στην ανάπτυξη του γυναικείου του Arabidopsis thaliana;
869	Μελέτη της σχέσης της αρτηριακής πίεσης ιατρείου, των μετρήσεων στο σπίτι και της περιπατητικής αρτηριακής πίεσης με την καρδιαγγειακή νοσηρότητα και θνητότητα ασθενών υπό περιτοναϊκή κάθαρση.
418	ΔΙΕΡΕΥΝΗΣΗ ΤΟΥ ΡΟΛΟΥ ΤΩΝ ΠΡΩΤΕΪΝΩΝ-ΠΟΛΥΚΥΣΤΙΝΩΝ ΣΤΑ ΝΕΟΠΛΑΣΜΑΤΙΚΑ ΚΥΤΤΑΡΑ ΓΛΟΙΩΜΑΤΩΝ
32	Συσχέτιση της γεωμετρίας του στεφανιαίου αρτηριακού δικτύου και κλινικών παραμέτρων με την κατανομή, πολυπλοκότητα και βαρύτητα της στεφανιαίας νόσου: μια πιλοτική μελέτη με τη χρήση πολυτομικής αξονικής στεφανιογραφίας
1490	Αρτηριακή πίεση και γνωσιακές λειτουργίες σε παιδιά και εφήβους
1990	Διερεύνηση βιοποικιλότητας παθογόνων, επικίνδυνων προς τη δημόσια υγεία, σε δίθυρα που διανέμονται από τις ιχθυαγορές του νομού Θεσσαλονίκης.
1522	microRNA από εξωσώματα Αρχέγονων Μεσεγχυματικών Κυττάρων του Μυελού των Οστών και διαταραχή της αιμοποίησης στα Μυελοδυσπλαστικά Σύνδρομα
528	Ο ρόλος του πυρηνικού υποδοχέα PPARβ/δ στην ομοιόσταση των μιτοχονδρίων των καρδιομυοκυττάρων σε ένα ζωικό πρότυπο καρδιακής ανεπάρκειας
306	Η σχέση αριθμού ειδών–επιφάνειας αλλιώς: εκτίμηση της ελάχιστης επιφάνειας που περιέχει S είδη
1030	"Διερεύνηση μακροχρόνιων επιπτώσεων στη ψυχική υγεία ασθενών μετά από επεισόδιο πνευμονικής εμβολής, εκτίμηση της ενσυναίσθησης και της συγχωρητικότητας"
100	In vitro μελέτη της αντικαρκινικής δράσης πολυφαινολών ελληνικού ελαιόλαδου
2053	Εξαγωγή βιοδεικτών από δεδομένα ψηφιακής απεικόνισης και μοριακής βιολογίας με χρήση υπολογιστικών μοντέλων για υποβοήθηση της διάγνωσης, πρόγνωσης και θεραπείας του κακοήθους μελανώματος
185	Πώς τα μιτοχόνδρια ρυθμίζουν το στρες; Ας ρωτήσουμε τη μεταβολομική
47	Ορθολογικός σχεδιασμός νέων παραγόντων κατά του ενζύμου ελικάσης του ιού του κίτρινου πυρετού με υπολογιστικές μεθόδους.

Τελικός βαθμός	Φορέας
98,3	ΑΡΙΣΤΟΤΕΛΕΙΟ ΠΑΝΕΠΙΣΤΗΜΙΟ ΘΕΣ/ΝΙΚΗΣ
97,8	ΠΑΝΕΠΙΣΤΗΜΙΟ ΙΩΑΝΝΙΝΩΝ
97,45	ΑΡΙΣΤΟΤΕΛΕΙΟ ΠΑΝΕΠΙΣΤΗΜΙΟ ΘΕΣ/ΝΙΚΗΣ
96,77	ΠΑΝΕΠΙΣΤΗΜΙΟ ΚΡΗΤΗΣ
96,65	ΠΑΝΕΠΙΣΤΗΜΙΟ ΚΡΗΤΗΣ
96,65	ΠΑΝΕΠΙΣΤΗΜΙΟ ΑΙΓΑΙΟΥ
96,52	ΠΑΝΕΠΙΣΤΗΜΙΟ ΠΑΤΡΩΝ
96,22	ΕΛΛΗΝΙΚΟ ΙΝΣΤΙΤΟΥΤΟ ΠΑΣΤΕΡ (Ε.Ι.Π.)
95,97	ΔΗΜΟΚΡΙΤΕΙΟ ΠΑΝΕΠΙΣΤΗΜΙΟ ΘΡΑΚΗΣ
95,9	ΙΔΡΥΜΑ ΤΕΧΝΟΛΟΓΙΑΣ & ΕΡΕΥΝΑΣ - ΙΤΕ
95,85	ΕΘΝΙΚΟ ΙΔΡΥΜΑ ΕΡΕΥΝΩΝ
95,82	ΠΑΝΕΠΙΣΤΗΜΙΟ ΠΑΤΡΩΝ
95,62	ΑΡΙΣΤΟΤΕΛΕΙΟ ΠΑΝΕΠΙΣΤΗΜΙΟ ΘΕΣ/ΝΙΚΗΣ
95,35	ΠΑΝΕΠΙΣΤΗΜΙΟ ΑΙΓΑΙΟΥ
95,12	ΙΔΡΥΜΑ ΙΑΤΡΟΒΙΟΛΟΓΙΚΩΝ ΕΡΕΥΝΩΝ ΑΚΑΔΗΜΙΑΣ ΑΘΗΝΩΝ (Ι.ΙΒ.Ε.Α.Α.)
95,07	ΠΑΝΕΠΙΣΤΗΜΙΟ ΚΡΗΤΗΣ
94,62	ΕΘΝΙΚΟ ΚΕΝΤΡΟ ΕΡΕΥΝΑΣ ΦΥΣΙΚΩΝ ΕΠΙΣΤΗΜΩΝ "ΔΗΜΟΚΡΙΤΟΣ"
94,52	ΔΗΜΟΚΡΙΤΕΙΟ ΠΑΝΕΠΙΣΤΗΜΙΟ ΘΡΑΚΗΣ
94,5	ΠΑΝΕΠΙΣΤΗΜΙΟ ΙΩΑΝΝΙΝΩΝ
94,47	ΠΑΝΕΠΙΣΤΗΜΙΟ ΙΩΑΝΝΙΝΩΝ
94,37	ΠΑΝΕΠΙΣΤΗΜΙΟ ΠΑΤΡΩΝ

94,2	ΑΡΙΣΤΟΤΕΛΕΙΟ ΠΑΝΕΠΙΣΤΗΜΙΟ ΘΕΣ/ΝΙΚΗΣ
93,97	ΕΘΝΙΚΟ & ΚΑΠΟΔΙΣΤΡΙΑΚΟ ΠΑΝΕΠΙΣΤΗΜΙΟ ΑΘΗΝΩΝ
93,87	ΠΑΝΕΠΙΣΤΗΜΙΟ ΘΕΣΣΑΛΙΑΣ
93,85	ΕΘΝΙΚΟ ΚΕΝΤΡΟ ΕΡΕΥΝΑΣ ΦΥΣΙΚΩΝ ΕΠΙΣΤΗΜΩΝ "ΔΗΜΟΚΡΙΤΟΣ"
93,75	ΕΘΝΙΚΟ ΚΕΝΤΡΟ ΕΡΕΥΝΑΣ ΦΥΣΙΚΩΝ ΕΠΙΣΤΗΜΩΝ "ΔΗΜΟΚΡΙΤΟΣ"
93,7	ΕΛΛΗΝΙΚΟ ΚΕΝΤΡΟ ΘΑΛΑΣΣΙΩΝ ΕΡΕΥΝΩΝ - ΕΛΚΕΘΕ
93,5	ΕΛΛΗΝΙΚΟ ΙΝΣΤΙΤΟΥΤΟ ΠΑΣΤΕΡ (Ε.Ι.Π.)
93,32	ΑΡΙΣΤΟΤΕΛΕΙΟ ΠΑΝΕΠΙΣΤΗΜΙΟ ΘΕΣ/ΝΙΚΗΣ
93,32	ΙΔΡΥΜΑ ΙΑΤΡΟΒΙΟΛΟΓΙΚΩΝ ΕΡΕΥΝΩΝ ΑΚΑΔΗΜΙΑΣ ΑΘΗΝΩΝ (Ι.ΙΒ.Ε.Α.Α.)
93,3	ΙΔΡΥΜΑ ΙΑΤΡΟΒΙΟΛΟΓΙΚΩΝ ΕΡΕΥΝΩΝ ΑΚΑΔΗΜΙΑΣ ΑΘΗΝΩΝ (Ι.ΙΒ.Ε.Α.Α.)
93,27	ΧΑΡΟΚΟΠΕΙΟ ΠΑΝΕΠΙΣΤΗΜΙΟ
93,15	ΕΘΝΙΚΟ & ΚΑΠΟΔΙΣΤΡΙΑΚΟ ΠΑΝΕΠΙΣΤΗΜΙΟ ΑΘΗΝΩΝ
93,12	ΧΑΡΟΚΟΠΕΙΟ ΠΑΝΕΠΙΣΤΗΜΙΟ
93,05	ΑΡΙΣΤΟΤΕΛΕΙΟ ΠΑΝΕΠΙΣΤΗΜΙΟ ΘΕΣ/ΝΙΚΗΣ
92,97	ΙΔΡΥΜΑ ΙΑΤΡΟΒΙΟΛΟΓΙΚΩΝ ΕΡΕΥΝΩΝ ΑΚΑΔΗΜΙΑΣ ΑΘΗΝΩΝ (Ι.ΙΒ.Ε.Α.Α.)
92,97	ΠΑΝΕΠΙΣΤΗΜΙΟ ΙΩΑΝΝΙΝΩΝ
92,87	ΑΡΙΣΤΟΤΕΛΕΙΟ ΠΑΝΕΠΙΣΤΗΜΙΟ ΘΕΣ/ΝΙΚΗΣ
92,85	ΑΡΙΣΤΟΤΕΛΕΙΟ ΠΑΝΕΠΙΣΤΗΜΙΟ ΘΕΣ/ΝΙΚΗΣ
92,85	ΙΔΡΥΜΑ ΤΕΧΝΟΛΟΓΙΑΣ & ΕΡΕΥΝΑΣ - ΙΤΕ
92,8	ΠΑΝΕΠΙΣΤΗΜΙΟ ΙΩΑΝΝΙΝΩΝ
92,65	ΠΑΝΕΠΙΣΤΗΜΙΟ ΘΕΣΣΑΛΙΑΣ

92,5	ΑΡΙΣΤΟΤΕΛΕΙΟ ΠΑΝΕΠΙΣΤΗΜΙΟ ΘΕΣ/ΝΙΚΗΣ
92,45	ΠΑΝΕΠΙΣΤΗΜΙΟ ΚΡΗΤΗΣ
92,45	ΠΑΝΕΠΙΣΤΗΜΙΟ ΙΩΑΝΝΙΝΩΝ
92,3	ΠΑΝΕΠΙΣΤΗΜΙΟ ΚΡΗΤΗΣ
92,3	ΕΘΝΙΚΟ & ΚΑΠΟΔΙΣΤΡΙΑΚΟ ΠΑΝΕΠΙΣΤΗΜΙΟ ΑΘΗΝΩΝ
92,15	ΠΑΝΕΠΙΣΤΗΜΙΟ ΑΙΓΑΙΟΥ
92,1	ΓΕΩΠΟΝΙΚΟ ΠΑΝΕΠΙΣΤΗΜΙΟ ΑΘΗΝΩΝ
92,07	ΔΗΜΟΚΡΙΤΕΙΟ ΠΑΝΕΠΙΣΤΗΜΙΟ ΘΡΑΚΗΣ
92,02	ΕΛΛΗΝΙΚΟ ΙΝΣΤΙΤΟΥΤΟ ΠΑΣΤΕΡ (Ε.Ι.Π.)
92	ΕΘΝΙΚΟ ΙΔΡΥΜΑ ΕΡΕΥΝΩΝ
91,92	ΠΑΝΕΠΙΣΤΗΜΙΟ ΘΕΣΣΑΛΙΑΣ
91,9	ΕΘΝΙΚΟ & ΚΑΠΟΔΙΣΤΡΙΑΚΟ ΠΑΝΕΠΙΣΤΗΜΙΟ ΑΘΗΝΩΝ
91,87	ΠΑΝΕΠΙΣΤΗΜΙΟ ΘΕΣΣΑΛΙΑΣ
91,82	ΕΛΛΗΝΙΚΟ ΙΔΡΥΜΑ ΒΑΣΙΚΗΣ ΒΙΟΛΟΓΙΚΗΣ ΕΡΕΥΝΑΣ "ΑΛΕΞΑΝΔΡΟΣ ΦΛΕΜΙΓΚ"
91,37	ΕΘΝΙΚΟ ΚΕΝΤΡΟ ΕΡΕΥΝΑΣ & ΤΕΧΝΟΛΟΓΙΚΗΣ ΑΝΑΠΤΥΞΗΣ – ΕΚΕΤΑ
91,37	ΕΘΝΙΚΟ ΙΔΡΥΜΑ ΕΡΕΥΝΩΝ
91,32	ΑΡΙΣΤΟΤΕΛΕΙΟ ΠΑΝΕΠΙΣΤΗΜΙΟ ΘΕΣ/ΝΙΚΗΣ
91,15	ΔΗΜΟΚΡΙΤΕΙΟ ΠΑΝΕΠΙΣΤΗΜΙΟ ΘΡΑΚΗΣ
91,07	ΕΘΝΙΚΟ & ΚΑΠΟΔΙΣΤΡΙΑΚΟ ΠΑΝΕΠΙΣΤΗΜΙΟ ΑΘΗΝΩΝ

91,02	ΑΡΙΣΤΟΤΕΛΕΙΟ ΠΑΝΕΠΙΣΤΗΜΙΟ ΘΕΣ/ΝΙΚΗΣ
91	ΑΡΙΣΤΟΤΕΛΕΙΟ ΠΑΝΕΠΙΣΤΗΜΙΟ ΘΕΣ/ΝΙΚΗΣ
90,97	ΙΔΡΥΜΑ ΙΑΤΡΟΒΙΟΛΟΓΙΚΩΝ ΕΡΕΥΝΩΝ ΑΚΑΔΗΜΙΑΣ ΑΘΗΝΩΝ (Ι.ΙΒ.Ε.Α.Α.)
90,82	ΕΘΝΙΚΟ ΚΕΝΤΡΟ ΕΡΕΥΝΑΣ ΦΥΣΙΚΩΝ ΕΠΙΣΤΗΜΩΝ "ΔΗΜΟΚΡΙΤΟΣ"
90,82	ΕΘΝΙΚΟ & ΚΑΠΟΔΙΣΤΡΙΑΚΟ ΠΑΝΕΠΙΣΤΗΜΙΟ ΑΘΗΝΩΝ
90,75	ΠΑΝΕΠΙΣΤΗΜΙΟ ΠΑΤΡΩΝ
90,75	ΕΘΝΙΚΟ & ΚΑΠΟΔΙΣΤΡΙΑΚΟ ΠΑΝΕΠΙΣΤΗΜΙΟ ΑΘΗΝΩΝ
**90,52**	**ΑΡΙΣΤΟΤΕΛΕΙΟ ΠΑΝΕΠΙΣΤΗΜΙΟ ΘΕΣ/ΝΙΚΗΣ**
90,47	ΕΘΝΙΚΟ & ΚΑΠΟΔΙΣΤΡΙΑΚΟ ΠΑΝΕΠΙΣΤΗΜΙΟ ΑΘΗΝΩΝ
90,3	ΑΡΙΣΤΟΤΕΛΕΙΟ ΠΑΝΕΠΙΣΤΗΜΙΟ ΘΕΣ/ΝΙΚΗΣ
90,3	ΑΡΙΣΤΟΤΕΛΕΙΟ ΠΑΝΕΠΙΣΤΗΜΙΟ ΘΕΣ/ΝΙΚΗΣ
90,17	ΔΗΜΟΚΡΙΤΕΙΟ ΠΑΝΕΠΙΣΤΗΜΙΟ ΘΡΑΚΗΣ
90,15	ΑΡΙΣΤΟΤΕΛΕΙΟ ΠΑΝΕΠΙΣΤΗΜΙΟ ΘΕΣ/ΝΙΚΗΣ
90,12	ΧΑΡΟΚΟΠΕΙΟ ΠΑΝΕΠΙΣΤΗΜΙΟ
90,1	ΑΡΙΣΤΟΤΕΛΕΙΟ ΠΑΝΕΠΙΣΤΗΜΙΟ ΘΕΣ/ΝΙΚΗΣ
90,07	ΑΡΙΣΤΟΤΕΛΕΙΟ ΠΑΝΕΠΙΣΤΗΜΙΟ ΘΕΣ/ΝΙΚΗΣ
90,05	ΑΡΙΣΤΟΤΕΛΕΙΟ ΠΑΝΕΠΙΣΤΗΜΙΟ ΘΕΣ/ΝΙΚΗΣ
90,02	ΠΑΝΕΠΙΣΤΗΜΙΟ ΑΙΓΑΙΟΥ
89,97	ΑΡΙΣΤΟΤΕΛΕΙΟ ΠΑΝΕΠΙΣΤΗΜΙΟ ΘΕΣ/ΝΙΚΗΣ
89,9	ΠΑΝΕΠΙΣΤΗΜΙΟ ΙΩΑΝΝΙΝΩΝ

89,87	ΠΑΝΕΠΙΣΤΗΜΙΟ ΚΡΗΤΗΣ
89,8	ΕΘΝΙΚΟ & ΚΑΠΟΔΙΣΤΡΙΑΚΟ ΠΑΝΕΠΙΣΤΗΜΙΟ ΑΘΗΝΩΝ
89,75	ΧΑΡΟΚΟΠΕΙΟ ΠΑΝΕΠΙΣΤΗΜΙΟ
89,72	ΔΗΜΟΚΡΙΤΕΙΟ ΠΑΝΕΠΙΣΤΗΜΙΟ ΘΡΑΚΗΣ
89,62	ΠΑΝΕΠΙΣΤΗΜΙΟ ΘΕΣΣΑΛΙΑΣ
89,57	ΠΑΝΕΠΙΣΤΗΜΙΟ ΚΡΗΤΗΣ
89,57	ΕΘΝΙΚΟ & ΚΑΠΟΔΙΣΤΡΙΑΚΟ ΠΑΝΕΠΙΣΤΗΜΙΟ ΑΘΗΝΩΝ
89,52	ΑΡΙΣΤΟΤΕΛΕΙΟ ΠΑΝΕΠΙΣΤΗΜΙΟ ΘΕΣ/ΝΙΚΗΣ
89,52	ΠΑΝΕΠΙΣΤΗΜΙΟ ΔΥΤΙΚΗΣ ΑΤΤΙΚΗΣ
89,5	ΧΑΡΟΚΟΠΕΙΟ ΠΑΝΕΠΙΣΤΗΜΙΟ
89,5	ΠΑΝΕΠΙΣΤΗΜΙΟ ΠΑΤΡΩΝ
89,35	ΓΕΩΠΟΝΙΚΟ ΠΑΝΕΠΙΣΤΗΜΙΟ ΑΘΗΝΩΝ
89,32	ΑΛΕΞΑΝΔΡΕΙΟ ΤΕΙ ΘΕΣΣΑΛΟΝΙΚΗΣ
89,32	ΔΗΜΟΚΡΙΤΕΙΟ ΠΑΝΕΠΙΣΤΗΜΙΟ ΘΡΑΚΗΣ
89,3	ΠΑΝΕΠΙΣΤΗΜΙΟ ΑΙΓΑΙΟΥ
89,3	ΑΡΙΣΤΟΤΕΛΕΙΟ ΠΑΝΕΠΙΣΤΗΜΙΟ ΘΕΣ/ΝΙΚΗΣ
89,25	ΙΔΡΥΜΑ ΙΑΤΡΟΒΙΟΛΟΓΙΚΩΝ ΕΡΕΥΝΩΝ ΑΚΑΔΗΜΙΑΣ ΑΘΗΝΩΝ (Ι.ΙΒ.Ε.Α.Α.)
89,2	ΠΑΝΕΠΙΣΤΗΜΙΟ ΘΕΣΣΑΛΙΑΣ
89,17	ΙΔΡΥΜΑ ΙΑΤΡΟΒΙΟΛΟΓΙΚΩΝ ΕΡΕΥΝΩΝ ΑΚΑΔΗΜΙΑΣ ΑΘΗΝΩΝ (Ι.ΙΒ.Ε.Α.Α.)
89,12	ΕΘΝΙΚΟ & ΚΑΠΟΔΙΣΤΡΙΑΚΟ ΠΑΝΕΠΙΣΤΗΜΙΟ ΑΘΗΝΩΝ

89	ΠΑΝΕΠΙΣΤΗΜΙΟ ΘΕΣΣΑΛΙΑΣ
88,97	ΧΑΡΟΚΟΠΕΙΟ ΠΑΝΕΠΙΣΤΗΜΙΟ
88,87	ΕΘΝΙΚΟ & ΚΑΠΟΔΙΣΤΡΙΑΚΟ ΠΑΝΕΠΙΣΤΗΜΙΟ ΑΘΗΝΩΝ
88,85	ΕΘΝΙΚΟ ΚΕΝΤΡΟ ΕΡΕΥΝΑΣ & ΤΕΧΝΟΛΟΓΙΚΗΣ ΑΝΑΠΤΥΞΗΣ – ΕΚΕΤΑ
88,82	ΠΑΝΕΠΙΣΤΗΜΙΟ ΙΩΑΝΝΙΝΩΝ
88,8	ΠΑΝΕΠΙΣΤΗΜΙΟ ΠΑΤΡΩΝ
88,72	ΕΘΝΙΚΟ & ΚΑΠΟΔΙΣΤΡΙΑΚΟ ΠΑΝΕΠΙΣΤΗΜΙΟ ΑΘΗΝΩΝ
88,72	ΔΗΜΟΚΡΙΤΕΙΟ ΠΑΝΕΠΙΣΤΗΜΙΟ ΘΡΑΚΗΣ
88,65	ΑΡΙΣΤΟΤΕΛΕΙΟ ΠΑΝΕΠΙΣΤΗΜΙΟ ΘΕΣ/ΝΙΚΗΣ
88,62	ΠΑΝΕΠΙΣΤΗΜΙΟ ΙΩΑΝΝΙΝΩΝ
88,57	ΠΑΝΤΕΙΟ ΠΑΝΕΠΙΣΤΗΜΙΟ ΚΟΙΝΩΝΙΚΩΝ & ΠΟΛΙΤΙΚΩΝ ΕΠΙΣΤΗΜΩΝ
88,4	ΕΘΝΙΚΟ ΚΕΝΤΡΟ ΕΡΕΥΝΑΣ ΦΥΣΙΚΩΝ ΕΠΙΣΤΗΜΩΝ "ΔΗΜΟΚΡΙΤΟΣ"
88,37	ΠΑΝΕΠΙΣΤΗΜΙΟ ΠΑΤΡΩΝ
88,32	ΠΑΝΕΠΙΣΤΗΜΙΟ ΘΕΣΣΑΛΙΑΣ
88,25	ΠΑΝΕΠΙΣΤΗΜΙΟ ΠΑΤΡΩΝ
88,12	ΑΡΙΣΤΟΤΕΛΕΙΟ ΠΑΝΕΠΙΣΤΗΜΙΟ ΘΕΣ/ΝΙΚΗΣ
88,12	ΠΑΝΕΠΙΣΤΗΜΙΟ ΘΕΣΣΑΛΙΑΣ
88	ΑΡΙΣΤΟΤΕΛΕΙΟ ΠΑΝΕΠΙΣΤΗΜΙΟ ΘΕΣ/ΝΙΚΗΣ

87,97	ΠΑΝΕΠΙΣΤΗΜΙΟ ΚΡΗΤΗΣ
87,97	ΠΑΝΕΠΙΣΤΗΜΙΟ ΘΕΣΣΑΛΙΑΣ
87,95	ΑΡΙΣΤΟΤΕΛΕΙΟ ΠΑΝΕΠΙΣΤΗΜΙΟ ΘΕΣ/ΝΙΚΗΣ
87,87	ΑΡΙΣΤΟΤΕΛΕΙΟ ΠΑΝΕΠΙΣΤΗΜΙΟ ΘΕΣ/ΝΙΚΗΣ
87,75	ΕΘΝΙΚΟ & ΚΑΠΟΔΙΣΤΡΙΑΚΟ ΠΑΝΕΠΙΣΤΗΜΙΟ ΑΘΗΝΩΝ
87,62	ΑΡΙΣΤΟΤΕΛΕΙΟ ΠΑΝΕΠΙΣΤΗΜΙΟ ΘΕΣ/ΝΙΚΗΣ
87,62	ΑΡΙΣΤΟΤΕΛΕΙΟ ΠΑΝΕΠΙΣΤΗΜΙΟ ΘΕΣ/ΝΙΚΗΣ
87,6	ΑΡΙΣΤΟΤΕΛΕΙΟ ΠΑΝΕΠΙΣΤΗΜΙΟ ΘΕΣ/ΝΙΚΗΣ
87,5	ΠΑΝΕΠΙΣΤΗΜΙΟ ΚΡΗΤΗΣ
87,47	ΑΡΙΣΤΟΤΕΛΕΙΟ ΠΑΝΕΠΙΣΤΗΜΙΟ ΘΕΣ/ΝΙΚΗΣ
87,45	ΑΡΙΣΤΟΤΕΛΕΙΟ ΠΑΝΕΠΙΣΤΗΜΙΟ ΘΕΣ/ΝΙΚΗΣ
87,42	ΤΕΙ ΘΕΣΣΑΛΙΑΣ
87,4	ΕΛΛΗΝΙΚΟ ΙΝΣΤΙΤΟΥΤΟ ΠΑΣΤΕΡ (Ε.Ι.Π.)
87,3	ΠΑΝΕΠΙΣΤΗΜΙΟ ΠΑΤΡΩΝ
88,25	ΠΑΝΕΠΙΣΤΗΜΙΟ ΙΩΑΝΝΙΝΩΝ
88,42	ΓΕΩΠΟΝΙΚΟ ΠΑΝΕΠΙΣΤΗΜΙΟ ΑΘΗΝΩΝ
